# Defence signalling marker gene responses to hormonal elicitation differ between roots and shoots

**DOI:** 10.1093/aobpla/ply031

**Published:** 2018-05-16

**Authors:** Galini V Papadopoulou, Anne Maedicke, Katharina Grosser, Nicole M van Dam, Ainhoa Martínez-Medina

**Affiliations:** 1 German Centre for Integrative Biodiversity Research (iDiv) Halle-Jena-Leipzig, Molecular Interaction Ecology, Deutscher Platz, Leipzig, Germany; 2 Friedrich Schiller University Jena, Institute of Biodiversity, Jena, Germany; 3 Radboud University, Molecular Interaction Ecology, Institute of Water and Wetland Research (IWWR), GL Nijmegen, The Netherlands

**Keywords:** *Brassica*, hormonal signalling, marker genes, phytohormones, plant defences

## Abstract

Phytohormones such as jasmonic acid (JA), salicylic acid (SA), ethylene (ET) and abscisic acid (ABA) play a key role in regulation of plant immune responses to different attackers. Extensive research over recent years has led to the identification of molecular markers for specific hormonal-regulated defence pathways. However, most of our current knowledge on the regulation of plant immunity derives from studies focused on above-ground organs, mainly on the model plant *Arabidopsis thaliana*. Therefore, it is unclear whether the paradigms based on experiments on above-ground organs are entirely transferable to roots. Here, we used the non-model plant *Brassica rapa* to study the regulation dynamics of hormonal-related marker genes in both roots and shoots. These markers were identified in *Arabidopsis* shoots after elicitation of the JA-, SA-, ET- or ABA-signalling pathways, and are commonly used to study induced responses. We assessed whether the regulation of those genes by hormonal elicitation differs between roots and shoots. To discern whether the differences in marker gene expression between roots and shoots are related to differences in hormone production or to differential responsiveness, we also measured actual hormone content in the treated tissue after elicitation. Our results show that some of the widely used markers did not show specific responsiveness to single hormone applications in *B. rapa*. We further found that hormonal elicitation led to different response patterns of the molecular markers in shoots and roots. Our results suggest that the regulation of some hormonal-related marker genes in *B. rapa* is organ specific and differs from the *Arabidopsis*-derived paradigms.

## Teaser

Plants responses to environmental stresses are regulated by signalling hormones. Plant scientists commonly use marker genes to study which signalling pathways are activated. However, these markers were designed and tested for shoot responses in *Arabidopsis*. We showed that in roots and shoots of *Brassica rapa* some of these commonly used molecular markers did not show specific responsiveness to single hormone applications. Moreover, the same marker gene may respond differently to hormone application in roots and shoots. These findings suggest that marker gene responses can be organ and species specific, and should be interpreted with caution.

## Introduction

Phytohormones such as jasmonic acid (JA), salicylic acid (SA), ethylene (ET) and abscisic acid (ABA) act as signalling molecules that regulate plant responses to insect herbivores, pathogens and beneficial microbes (reviewed by Erb and Glauser 2010; Robert-Seilaniantz *et al.* 2011; Pieterse *et al.* 2012; Broekgaarden *et al.* 2015). Jasmonic acid is a key regulator of plant defences to necrotrophic pathogens, chewing insects and wound responses, whereas SA is mainly induced in response to biotrophic pathogens and phloem-sucking insects (Zarate *et al.* 2007; Pieterse *et al.* 2012). While the JA and SA signalling pathways (hereafter referred to as pathways) form the backbone of the plant’s immune system, ABA and ET play a more modulatory role. In the model plant *Arabidopsis thaliana* (hereafter *Arabidopsis*), the JA pathway consists of two distinct and antagonistic branches, the MYC- and the ERF-branch, which are co-regulated by ABA and ET, respectively. The ET-regulated ERF-branch of the JA pathway is associated with plant defences against necrotrophic pathogens, while the ABA-regulated MYC-branch is associated with wounding and insect herbivory (Anderson *et al.* 2004; Lorenzo and Solano 2005; Pré *et al.* 2008; Verhage *et al.* 2011; Kazan and Manners 2013). Antagonistic and synergistic interactions between pathways are well known, with the antagonistic interactions between JA and SA pathways being the most intensively studied (Berrocal‐Lobo *et al.* 2002; Leon-Reyes *et al.* 2009; Vos *et al.* 2015). These interconnections between hormonal pathways are known as ‘crosstalk’ and provide plants with a complex network that allows them to fine-tune their defences against different stimuli (Pieterse *et al.* 2012).

To link particular pathways with actual defence responses, molecular tools such as qPCR allow us to use the expression of several marker genes as indicators of the activation of specific pathways. For example, in *Arabidopsis PATHOGENESIS*-*RELATED PROTEIN 1* (*PR1*) is used as a marker gene for the SA pathway (Gaffney *et al.* 1993; van Wees *et al.* 1999; van Loon *et al.* 2006); the basic helix-loop-helix leucine zipper transcription factor *MYC2* and the *VEGETATIVE STORAGE PROTEIN 2* (*VSP2*) are used as markers for the ABA-modulated branch of JA pathway (Anderson *et al.* 2004; Lorenzo *et al.* 2004; Dombrecht *et al.* 2007), while the *ETHYLENE RESPONSE FACTOR 1* (*ERF1*) and *PLANT DEFENSIN 1.2* (*PDF1.2*) are commonly used as markers for the ET-modulated branch of the JA pathway (Penninckx *et al.* 1998; Lorenzo *et al.* 2003; Robert-Seilaniantz *et al.* 2011). In analogy, *ERF1* and the *ETHYLENE RECEPTOR 1* (*ETR1*) genes are used as markers for the ET pathway (Lorenzo *et al.* 2003; Lorenzo *et al.* 2004) and *LATE EMBRYOGENESIS ABUDANT 4* (*LEA4*) as a marker for the ABA pathway (Hoth *et al.* 2002; Hundertmark and Hincha 2008).

The vast majority of studies on the identification and characterization of marker genes for specific pathways has been done on the shoot tissues of the model plant *Arabidopsis*. Marker genes have been validated in *Arabidopsis* shoots by analysing their expression patterns after exogenous application of single or combined phytohormone solutions (Hoth *et al.* 2002). The information on marker gene responsiveness and the interactions between different pathways obtained in *Arabidopsis* shoots has been implemented in other plant species, including species of the closely related genus *Brassica* (Wang *et al.* 2011). In some *Brassica* spp., the responsiveness of several *Arabidopsis*-derived marker genes to exogenous hormonal application has been tested. For example, *VSP2* is up-regulated in response to JA application in *Brassica juncea* and *B. olearacea* (Mathur *et al.* 2013; Tytgat *et al.* 2013), *PR1* is responsive to SA application in *B. rapa* (Abe *et al.* 2011; Lee and Hong 2015) and *BnLEA4-1* is an ABA-responsive gene in *B. napus*, *B. juncea* and *B. carinata* (Dalal *et al.* 2009). Although *Arabidopsis* and *Brassica* spp. belong to the same family, marker gene responsiveness to activation of specific hormonal pathways might show important differences (Tytgat *et al.* 2013). Since an enormous amount of studies on plant immune responses to above-ground organisms is being conducted on *Brassica* spp., further validation of the *Arabidopsis*-derived marker genes in *Brassica* spp. is required (Soler *et al.* 2012; Maag *et al.* 2014; Kroes *et al.* 2016; Pineda *et al.* 2017).

Over the last 15 to 20 years, interest in the regulation of root-induced responses and how they affect above-ground defence responses has increased (van Dam *et al.* 2003; Papadopoulou and van Dam 2017). In natural environments, plant roots interact with a variety of organisms present in the rhizosphere (van der Putten 2003). It may therefore be expected that roots have an equally extensive signalling network as shoots for managing the diversity of below-ground interactions. Surprisingly, limited information on hormonal signalling and marker gene responsiveness is available for the root tissues of *Arabidopsis* or other plant species (Lawrence *et al.* 2012; Tytgat *et al.* 2013; Lu *et al.* 2015). A few available studies support the notion that transcriptional responses to hormonal elicitations in shoots and roots on the same plant may considerably differ (Lawrence *et al.* 2012; Tytgat *et al.* 2013). These findings raise the question whether the paradigms for hormonal signalling and the responses of the main marker genes as observed in shoots can be simply transferred to the roots of the same or different plant species. Considering the increasing research interest in plant immune responses to below-ground organisms (Barr *et al.* 2010; Martínez‐Medina *et al.* 2017a, b; Tsunoda *et al.* 2017), it is imperative to investigate hormonal signalling pathways regulating defence responses in root organs. To do so, it is also important to identify marker genes appropriate for roots. At present, most marker genes have been developed for shoot organs and may thus be unreliable for root studies.

In this study, we first tested whether genes known as markers for the main defence-related hormonal pathways (i.e. JA, SA, ABA and ET) in the *Arabidopsis*-shoot model are regulated similarly by hormonal elicitation in *B. rapa* shoots. The complete genome sequence of *B. rapa* is available (http://brassicadb.org). It shares 93 % of its gene families with *Arabidopsis* (Wang *et al.* 2011), which makes it a good model plant among *Brassica* spp. for molecular and genomic studies. Then, we investigated whether the regulation of the molecular markers after hormonal elicitation is similar in shoot and in root organs of *B. rapa*. Based on the great homology between *Brassica* and *Arabidopsis*, we expect that the regulation of the tested marker genes by hormonal elicitation in *B. rapa* shoots will be similar to that of *Arabidopsis* shoots. However, we expect differential regulation in root and shoot tissues of *B. rapa* (Tytgat *et al.* 2013). To test these assumptions, we analysed the expression patterns of *VSP2*, *PR1* and *ERF1* over time (from 4 to 48 h) in *B. rapa* shoots and roots after local elicitation with either methyl jasmonate (MeJA), ABA, SA or ethephon, an ET-releasing compound. Furthermore, we analysed the regulation of the predicted *B. rapa* 18 kDa seed maturation protein-like (hereafter referred to as *BrLEA4*) as a novel putative marker gene for ABA pathway in *B. rapa* shoots and roots. *BrLEA4* was selected as a homolog of the *Arabidopsis LEA4-5* gene, previously shown to be highly ABA responsive in vegetative tissues (Hoth *et al.* 2002). Moreover *LEA* genes are also responsive to ABA in soybean, tomato and maize (Martínez-Medina *et al.* 2013; Fernández *et al.* 2014; Zamora-Briseño and de Jiménez 2016). Furthermore, to link changes in marker gene expression with that in the accumulation of the phytohormones themselves, we also measured phytohormone levels in the same tissues. In this way, we could discern whether discrepancies in marker gene expression between roots and shoots were attributed to differences in actual hormonal levels in the treated tissues or to different marker gene responsiveness. We found that marker gene responsiveness to specific hormonal pathways in *B. rapa* deviated to some extent from those of the *Arabidopsis*-shoot model. In addition, we found that the same marker genes show differential regulation depending on the plant organ. Overall, our findings indicate that plant species and the specific plant organ should be considered in marker gene selection when studying regulation of plant defence responses.

## Methods

### Plant growth

*Brassica rapa* seeds, originating from a wild population (Maarsen, The Netherlands), were germinated on glass beads in plastic containers closed with a transparent plastic lid. The containers were kept for 1 week in a climate chamber (Percival Scientific, Perry, IA, USA) at 20 °C, with a 16-h light and 8-h dark cycle, 60 % relative humidity and 50 µmol m^−2^ s^−1^ photosynthetic active radiation. Seedlings were then transplanted in 11 × 11 × 12 cm pots, filled with 1:1 mixture of potting soil (Floradur B Pot Clay Medium, Floragard Vertriebs GmbH, Oldenburg, Germany) and sand (Gerhard Rösl GmbH, Jesewitz OT Liemehna, Germany). During the transplantation plants were fertilized with the Osmocote®Pro 3-4M slow-release fertilizer (Everris International B.V., The Netherlands). The plants were grown in a greenhouse with a 16-h light (27 °C) and 8-h dark (21 °C) cycle at 50 % relative humidity, and watered as needed. Four weeks after seed germination, the plants that had five fully expanded leaves were used for the experiments.

### Hormonal application

The roots or shoots of *B. rapa* plants were either treated with 100 µM MeJA (Sigma-Aldrich, Munich, Germany), 1 mM SA (Carl Roth, Karlsruhe, Germany), 10 µM ABA (Sigma-Aldrich, Munich, Germany) or 7 mM ethephon (2-chloroethylphosphonic acid, Sigma-Aldrich, Munich, Germany). Hormone solutions were amended with 0.015 % (v/v) Silwet L77 (Van Meeuwen Chemicals BV, Weesp, The Netherlands). Control plants were treated with a water solution containing 0.015 % Silwet L77.

Different groups of plants were used for root and shoot treatments. Shoot treatment was performed by applying 1 mL of MeJA, ABA, SA, ethephon or control solution to the upper adaxial side of the fourth fully expanded leaf (counted from the soil). In case of MeJA, ABA, SA and the respective control plants, 1 mL was also applied to the lower, adaxial surface of the same leaf. Root treatment was performed by applying 50 mL of MeJA, ABA, SA, ethephon or control solutions to the saucers from which roots quickly absorbed the solution. To avoid ethephon evaporating into the surrounding air, ethephon-treated plants were covered with transparent foil. As control plants for the ethephon treatment, a separated set of plants treated with control solution was used, which was also covered with a transparent foil. The plants were harvested at 4, 8, 24 or 48 h after hormonal application and five biological replicates (single plants) per time point were used. Roots were carefully washed to remove the adherent soil. The roots of root-treated plants and the local leaf of the shoot-treated plants were harvested, immediately frozen in liquid nitrogen and stored at −80 °C.

### Quantitative RT–PCR analysis

Total RNA was extracted from ~100 mg of ground plant tissue using innuPREP Plant RNA Kit (Analytik Jena, Jena, Germany) and treated with DNase I (Biozym Scientific, Hessisch Oldendorf, Germany) following the manufacturers’ instructions. For each sample, 1 µg of purified total RNA was subjected to reverse transcription using oligo(dT)_20_ primer and RevertAid H Minus enzyme (Thermo Scientific, Waltham, MA, USA) following the manufacturer’s instructions. Undiluted cDNA was used for real-time quantitative RT–PCR (qPCR) analyses of *PR1* and *VSP2* in root samples. For the rest of the qPCR analyses 10-fold diluted cDNA was used. qPCRs were performed by using SYBR Green qPCR Master Mix (2×) (Thermo Scientific, Waltham, MA, USA) according to the manufacturer’s instructions. All the qPCRs were run in 96-well plates with a PikoReal 96 instrument (Thermo Scientific, Waltham, MA, USA) under the following conditions: incubation at 50 °C for 2 min and 95 °C for 5 min, followed by 40 cycles of incubation at 95 °C for 30 s, 58 °C for 30 s and 72 °C for 30 s. Relative quantification of mRNA levels was performed using the comparative 2^−ΔCT^ method (Livak and Schmittgen 2001). Expression values were normalized by using the housekeeping gene *TIP41* (Chen *et al.* 2010; Chandna *et al.* 2012). Gene-specific primers listed in Table 1 were used for qPCR analysis. For *BrLEA4* primer design, a nucleotide sequence of *Arabidopsis LEA4-5* (AT5G06760) was subjected to NCBI nBLAST tool (https://blast.ncbi.nlm.nih.gov/Blast.cgi). The *B. rapa* sequence (LOC103855696) predicted to encode for 18 kDa seed maturation protein-like (referred here as *BrLEA4*) showed 100 % similarity with the *Arabidopsis LEA4-5* sequence and was selected for a primer design. Primers were designed using the Primer3 tool (v. 0.4.0, http://bioinfo.ut.ee/primer3-0.4.0/). The specificity and efficiency of all primers used in this study were tested. Primer specificity was tested with agarose gel electrophoresis and melting curve analysis following qPCRs. Primers resulting in a single product were selected. PCRs were performed by using GoTaq® DNA Polymerase (Promega, Madison, WI, USA), according to manufacturer’s instructions on a Techne® Prime Elite thermal cycler (Bibby Scientific Ltd, Stone, UK). PCRs were run for 95 °C for 2 min, 35 cycles of incubation for 95 °C for 30 s, 58 °C for 30 s and 72 °C for 45 s, followed by 72 °C for 5 min. In order to determine the gene-specific PCR efficiency, a 10-fold serial dilutions of cDNA were used to generate a standard curve. The correlation coefficient (*R*^2^) and the PCR efficiency were calculated by using the slopes of the standard curves **[see Supporting Information—Fig. S1]**. The linear *R*^2^ for all the primers ranged from 0.938 to 0.999 over 100-fold of cDNA dilution.

**Table 1.** Gene information and primers sequences used for gene expression analysis. n.a.: not available.

Gene name	Accession number	*Arabidopsis thaliana* locus	Sequence (5′-3′)	Marker for	Reference
*VSP2*	Bra020470	AT5G24770	F: TCTACGCCAAAGGACTTGCT	JA pathway	T. O. Tytgat (unpubl. data)
			R: CCCGTATCCATATTGAGCGTA		
*PR1*	n.a.	AT2G14610	F: CTACGCCGACCGACTAAGAG	SA pathway	Mathur *et al.* (2013)
			R: CTACTCCCGGCCAAGTTCTC		
*ERF1*	Bra023744 Bra023746	AT3G23240	F: CGGCGGAGAGAGTTAAAGAG	ET pathway	Mathur *et al.* (2013)
			R: AACACCCATCCTCGTAGCTG		
*BrLEA4*	Bra005911	AT5G06760	F: TCAGCCACTCACTCAACCAC	ABA pathway	Present study
			R: GTCCGACCAGTTCCAGTGTT		
*TIP41*	Bra011516	AT4G34270	F: TGCGAAAGGGTATCCAGTTG	Housekeeping gene	T. O. Tytgat (unpubl. data)
			R: ATCACCGGAAGCCTCTGAC		

### Phytohormone measurements

Phytohormone extraction and purification were performed as described by Machado *et al.* (2013) with some modifications. Briefly, shoot and root tissue (50–100 mg per sample) was extracted with 1 mL ethyl acetate containing 40 ng of each of the following internal phytohormone standards: D_6_-ABA, D_6_-SA (OlChemIm Ltd, Olomouc, Czech Republic) and D_6_-JA (HPC Standards GmbH, Borsdorf, Germany). Samples were vortexed for 10 min, centrifuged at 14000 rpm for 2 min at 4 °C and the supernatants were evaporated until dryness in a Speed-Vac (Labconco, USA) at room temperature. Pellets were then suspended in 200 µL methanol:water (70:30) and dissolved using a Fisherbrand FB 15061 ultrasonic bath (Fisher Scientific, UK) for 15 min. Phytohormones were analysed using liquid chromatography (Bruker Advance UHPLC, Bremen, Germany) coupled to mass spectrometer (Bruker Elite EvoQ Triple quad, Bremen, Germany) (LC-MS), as described by Schäfer *et al.* (2016). Separation was achieved on a Zorbax Eclipse XDB-C 18 column (50 × 4.6 mm, 1.8 mm; Agilent Technologies, Boeblingen, Germany) with 0.05 % formic acid in water and 0.05 % formic acid in acetonitrile as mobile phases A and B, respectively. Samples were analysed in a randomized sequence including acetonitrile samples in between as controls. Data acquisition and processing were performed using the ‘MS data Review’ software (Bruker MS Workstation, version 8.2). Phytohormone levels were calculated over the amount of fresh mass of plant material (ng^−1^ mg^−1^ fresh mass).

### Statistical analysis

Gene expression and phytohormone level data were log transformed to meet the assumptions of normality and homogeneity of variances and then subjected to two-way ANOVA (R software, version 3.1.2). The data were analysed per treatment using a model containing treatment (control, hormonal application), time (4, 8, 24, 48 h) and their interaction term as factors. Following two-way ANOVA, one-way ANOVA was performed for each time point to analyse the effect of hormonal application when time point had a significant effect. Tukey test was performed on the interaction effect (treatment × time), when the interaction was significant. Phytohormone level data were corrected for instrument carry over by subtracting the average value found in acetonitrile control samples from the experimental-samples data. Following subtraction, negative values were replaced with 0.00001 for data analysis and visualization. For the visualization of data, fold changes (FC) in gene expression and phytohormone levels were calculated by dividing the normalized expression or phytohormone levels **[see Supporting Information—Tables S1–S3]** of each treated plant by the average expression or phytohormone levels of the respective control group. The data obtained from the MeJA, ABA and SA experiments were also analysed for the overall treatment effect with a two-way ANOVA model containing control, MeJA, SA and ABA as treatment term **[see Supporting Information—Tables S4 and S5]**.

## Results

### Effect of hormonal application on marker gene expression

**Figure 1.**
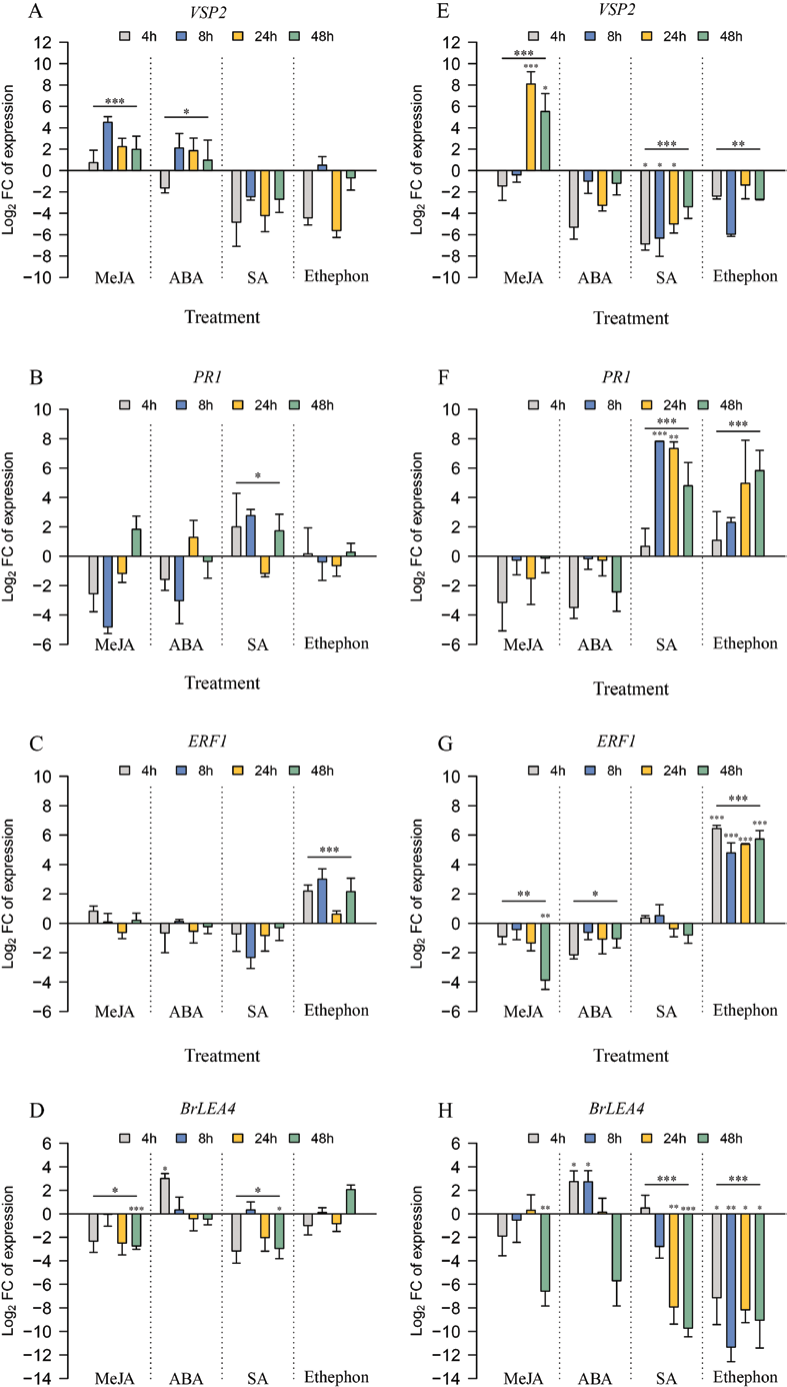
Relative expression of the hormonal-related marker genes in *Brassica rapa* shoots (left column, panels A–D) and roots (right column, panels E–H) in response to hormonal application. Expression levels of (A, E) *VSP2*, (B, F) *PR1*, (C, G) *ERF1* and (D, H) *BrLEA4* were measured at 4, 8, 24 and 48 h after local MeJA, ABA, SA or ethephon application. Data were normalized over the housekeeping gene *TIP41*, and are represented as mean log_2_ fold changes (log_2_ FC + SE) in relation to the respective control. In each hormonal treatment, asterisks over the horizontal line represent the overall significant treatment main effect and those over individual bars indicate significant differences between the treatment group and their respective control plants, according to two-way ANOVA (*n* = 3–4 per treatment and harvest time) *P ≤ 0.05; **P ≤ 0.01; ***P ≤ 0.001.

Gene expression analysis in *B. rapa* shoots showed that irrespective of the time point after treatment, *VSP2* expression was significantly up-regulated by MeJA and ABA application compared to control plants (treatment effect, *F*_1, 24_ = 18.36, *P* < 0.001 and *F*_1, 24_ = 5.68, *P* = 0.025, respectively, Table 2, Fig. 1A). Salicylic acid and ethephon application did not have a significant effect on *VSP2* expression, though *VSP2* tended to be down-regulated by SA treatment (treatment effect, *F*_1, 24_ = 3.00, *P* = 0.096, Table 2, Fig. 1A), as well as by ethephon 4 and 24 h after application (interaction effect, *F*_3, 24_ = 2.48, *P* = 0.085, Table 2, Fig. 1A). In *B. rapa* roots, *VSP2* expression was significantly up-regulated specifically in response to MeJA application. The effect of MeJA application on *VSP2* expression was time-dependent (interaction effect, *F*_3, 22_ = 6.07, *P* = 0.004, Table 3), with statistically significant up-regulation at 24 and 48 h after treatment (Tukey *post hoc* test, *P* = 0.001 and *P* = 0.029, respectively, Fig. 1E). By contrast, SA and ethephon application significantly down-regulated *VSP2* expression (two-way ANOVA, Table 3, Fig. 1E). Although ABA application had no significant effect on *VSP2* expression, there was a trend for down-regulation of this gene in ABA-treated roots (treatment effect, *F*_1, 19_ = 3.46, *P* = 0.078, Table 3).

**Table 2.** Statistical analyses (*F*- and *P*-values) of the effects of local hormonal application on gene expression levels in *Brassica rapa* shoots. The expression levels of *VSP2*, *PR1*, *ERF1* and *BrLEA4* were measured in *B. rapa* shoots after MeJA, ABA, SA or ethephon application to the shoots (*n* = 3–4 per treatment and harvest time). The data were analysed per hormonal treatment group using a two-way ANOVA model containing treatment (control, hormonal application), time (4, 8, 24, 48 h) and their interaction term as factors. Statistically significant effects (*P* ≤ 0.05) are indicated in bold.

Treatment	Factor	Gene			
		*VSP2*	*PR1*	*ERF1*	*BrLEA4*
MeJA	Treatment (1)	***F***_**1, 24**_ **= 18.36, *P* < 0.001**	*F*_1, 22_ = 0.24, *P* = 0.63	*F*_1, 24_ = 0.95, *P* = 0.34	***F***_**1, 22**_ **= 5.02, *P* = 0.035**
	Time (2)	*F*_3, 24_ = 2.18, *P* = 0.117	*F*_3, 22_ = 0.51, *P* = 0.679	*F*_3, 24_ = 0.97, *P* = 0.425	***F***_**3, 22**_ **= 6.17, *P* = 0.003**
	Interaction (1.2)	*F*_3, 24_ = 0.88, *P* = 0.465	*F*_3, 22_ = 1.94, *P* = 0.152	*F*_3, 24_ = 1.1, *P* = 0.37	*F*_3, 22_ = 1.09, *P* = 0.373
ABA	Treatment (1)	***F***_**1, 24**_ **= 5.68, *P* = 0.025**	*F*_1, 22_ = 0.16, *P* = 0.692	*F*_1, 22_ = 0.12, *P* = 0.73	*F*_1, 20_ = 3.83, *P* = 0.064
	Time (2)	*F*_3, 24_ = 1.33, *P* = 0.287	*F*_3, 22_ = 1.11, *P* = 0.367	*F*_3, 22_ = 0.98, *P* = 0.42	***F***_**3, 20**_ **= 11.8, *P* < 0.001**
	Interaction (1.2)	*F*_3, 24_ = 0.25, *P* = 0.863	*F*_3, 22_ = 1.23, *P* = 0.323	*F*_3, 22_ = 0.05, *P* = 0.986	***F***_**3, 20**_ **= 3.31, *P* = 0.041**
SA	Treatment (1)	*F*_1, 24_ = 3.00, *P* = 0.096	***F***_**1, 23**_ **= 5.82, *P* = 0.024**	*F*_1, 24_ = 2.43, *P* = 0.133	***F***_**1, 21**_ **= 4.31, *P* = 0.05**
	Time (2)	*F*_3, 24_ = 0.79, *P* = 0.512	***F***_**3, 23**_ **= 3.58, *P* = 0.03**	*F*_3, 24_ = 1.88, *P* = 0.16	***F***_**3, 21**_ **= 5.31, *P* = 0.007**
	Interaction (1.2)	*F*_3, 24_ = 0.41, *P* = 0.745	*F*_3, 23_ = 0.43, *P* = 0.736	*F*_3, 24_ = 0.83, *P* = 0.492	*F*_3, 21_ = 1.09, *P* = 0.376
Ethephon	Treatment (1)	*F*_1, 24_ = 1.46, *P* = 0.239	*F*_1, 24_ = 0.92, *P* = 0.346	***F***_**1, 22**_ **= 29.16, *P* < 0.001**	*F*_1, 24_ = 1.89, *P* = 0.183
	Time (2)	*F*_3, 24_ = 2.43, *P* = 0.090	*F*_3, 24_ = 0.38, *P* = 0.766	*F*_3, 22_ = 1.94, *P* = 0.153	*F*_3, 24_ = 2.27, *P* = 0.106
	Interaction (1.2)	*F*_3, 24_ = 2.48, *P* = 0.085	*F*_3, 24_ = 0.06, *P* = 0.979	*F*_3, 22_ = 1.42, *P* = 0.262	*F*_3, 24_ = 2.24, *P* = 0.11

**Table 3.** Statistical analyses (*F*- and *P*-values) of the effects of local hormonal application on gene expression levels in *Brassica rapa* roots. The expression levels of *VSP2*, *PR1*, *ERF1* and *BrLEA4* were measured in *B. rapa* roots after MeJA, ABA, SA or ethephon application to the roots (*n* = 3–4 per treatment and harvest time). The data were analysed per hormonal treatment group using a two-way ANOVA model containing treatment (control, hormonal application), time (4, 8, 24, 48 h) and their interaction term as factors. Statistically significant effects (*P* ≤ 0.05) are indicated in bold.

Treatment	Factor	Gene			
		*VSP2*	*PR1*	*ERF1*	*BrLEA4*
MeJA	Treatment (1)	***F***_**1, 22**_ **= 22.57, *P* < 0.001**	*F*_1, 22_ = 0.00, *P* = 0.964	***F***_**1, 19**_ **= 12.84, *P* = 0.002**	*F*_1, 23_ = 0.88, *P* = 0.358
	Time (2)	*F*_3, 22_ = 2.19, *P* = 0.117	*F*_3, 22_ = 0.57, *P* = 0.639	***F***_**3, 19**_ **= 12.16, *P* < 0.001**	***F***_**3, 23**_ **= 4.61, *P* = 0.011**
	Interaction (1.2)	***F***_**3, 22**_ **= 6.07, *P* = 0.004**	*F*_3, 22_ = 0.36, *P* = 0.786	***F***_**3, 19**_ **= 4.34, *P* = 0.017**	***F***_**3, 23**_ **= 3.24, *P* = 0.04**
ABA	Treatment (1)	*F*_1, 19_ = 3.46, *P* = 0.078	*F*_1, 22_ = 0.13, *P* = 0.722	***F***_**1, 20**_ **= 6.72, *P* = 0.017**	*F*_1, 22_ = 2.5, *P* = 0.128
	Time (2)	***F***_**3, 19**_ **= 8.2, *P* = 0.001**	*F*_3, 22_ = 0.50, *P* = 0.684	*F*_3, 20_ = 3, *P* = 0.06	***F***_**3, 22**_ **= 4.03, *P* = 0.02**
	Interaction (1.2)	*F*_3, 19_ = 0.59, *P* = 0.626	*F*_3, 22_ = 1.23, *P* = 0.322	*F*_3, 20_ = 0.33, *P* = 0.805	***F***_**3, 22**_ **= 6.02, *P* = 0.004**
SA	Treatment (1)	***F***_**1, 20**_ **= 25.4, *P* < 0.001**	***F***_**1, 20**_ **= 45.69, *P* < 0.001**	*F*_1, 18_ = 0.02, *P* = 0.888	***F***_**1, 23**_ **= 21.68, *P* < 0.001**
	Time (2)	***F***_**3, 20**_ **= 5.51, *P* = 0.006**	*F*_3, 20_ = 1.31, *P* = 0.299	***F***_**3, 18**_ **= 8.25, *P* = 0.001**	***F***_**3, 23**_ **= 8.75, *P* < 0.001**
	Interaction (1.2)	*F*_3, 20_ = 0.75, *P* = 0.538	***F***_**3, 20**_ **= 3.56, *P* = 0.033**	*F*_3, 18_ = 0.94, *P* = 0.444	***F***_**3, 23**_ **= 9.5, *P* < 0.001**
Ethephon	Treatment (1)	***F***_**1, 18**_ **= 9.62, *P* = 0.006**	***F***_**1, 22**_ **= 14.97, *P* = 0.001**	***F***_**1, 20**_ **= 234.81, *P* < 0.001**	***F***_**1, 24**_ **= 43.49, *P* < 0.001**
	Time (2)	*F*_3, 18_ = 0.97, *P* = 0.43	*F*_3, 22_ = 0.63, *P* = 0.606	***F***_**3, 20**_ **= 5.54, *P* = 0.006**	***F***_**3, 24**_ **= 4.45, *P* = 0.013**
	Interaction (1.2)	*F*_3, 18_ = 1.65, *P* = 0.213	*F*_3, 22_ = 1.15, *P* = 0.35	*F*_3, 20_ = 0.72, *P* = 0.549	*F*_3, 24_ = 0.81, *P* = 0.503

*PR1* expression was significantly up-regulated in SA-treated shoots compared to control (treatment effect, *F*_1, 23_ = 5.82, *P* = 0.024, Table 2, Fig. 1B). Methyl jasmonate, ABA or ethephon application had no significant effect on *PR1* expression (treatment effect, *F*_1, 22_ = 0.24, *P* = 0.63; *F*_1, 22_ = 0.16, *P* = 0.692 and *F*_1, 24_ = 0.92, *P* = 0.346, respectively, Table 2, Fig. 1B). In the roots, expression of *PR1* was strongly up-regulated in response to SA application (treatment effect, *F*_1, 20_ = 45.69, *P* < 0.001, Table 3, Fig. 1F). The effect of SA treatment on *PR1* expression in the roots was time-dependent (interaction effect, *F*_3, 20_ = 3.56, *P* = 0.033, Table 3), with a significant up-regulation at 8 and 24 h after application compared to control plants (Tukey *post hoc* test, 8 h: *P* = 0.001; 24 h: *P* = 0.002). Interestingly, ethephon application also up-regulated the expression of *PR1* in roots, irrespective of the time after treatment (treatment effect, *F*_1, 22_ = 14.97, *P* = 0.001; interaction effect, *F*_3, 22_ = 1.15, *P* = 0.35, Table 3, Fig. 1F). Methyl jasmonate nor ABA application significantly affected *PR1* expression in roots (treatment effect, *F*_1, 22_ = 0.00, *P* = 0.964 and *F*_1, 22_ = 0.13, *P* = 0.722, respectively, Table 3, Fig. 1F).

In the shoots, expression of *ERF1* was significantly up-regulated by ethephon treatment (treatment effect, *F*_1, 22_ = 29.16, *P* < 0.001, Table 2, Fig. 1C). Application of MeJA, ABA or SA had no significant effect on *ERF1* shoot expression (treatment effect, *F*_1, 24_ = 0.95, *P* = 0.34; *F*_1, 22_ = 0.12, *P* = 0.73 and *F*_1, 24_ = 2.43, *P* = 0.133, respectively, Table 2, Fig. 1C). Also in the roots, *ERF1* was significantly up-regulated by ethephon application (treatment effect, *F*_1, 20_ = 234.81, *P* < 0.001, Table 3, Fig. 1G). Interestingly, local *ERF1* expression was significantly down-regulated by MeJA and ABA application to the roots (treatment effect, *F*_1, 19_ = 12.84, *P* = 0.002 and *F*_1, 20_ = 6.72, *P* = 0.017, respectively, Table 3, Fig. 1G). The down-regulation of *ERF1* by MeJA was time-dependent (interaction effect, *F*_3, 19_ = 4.34, *P* = 0.017, Table 3, Fig. 1G) and was significant 48 h after the treatment (Tukey *post hoc* test, 48 h: *P* = 0.002). Application of SA did not significantly affect *ERF1* expression in roots (treatment effect, *F*_1, 18_ = 0.02, *P* = 0.888, Table 3).

In shoots, the overall expression of *BrLEA4* was not significantly affected by ABA treatment, though there was a trend for up-regulation (treatment effect, *F*_1, 20_ = 3.83, *P* = 0.064, Table 2, Fig. 1D). Although *BrLEA4* was specifically up-regulated at 4 h after ABA application (one-way ANOVA, *F*_1, 6_ = 7.92, *P* = 0.031), at later time points, *BrLEA4* expression returned to basal levels. Shoot treatment with ethephon did not have any significant effect on the *BrLEA4* expression (treatment effect, *F*_1, 24_ = 1.89, *P* = 0.183, Table 2, Fig. 1D). However, MeJA and SA treatments significantly down-regulated the expression of this gene in *B. rapa* shoots (two-way ANOVA, Table 2, Fig. 1D). In *B. rapa* roots, expression of *BrLEA4* was significantly up-regulated by ABA treatment at 4 and 8 h after application (one-way ANOVA, 4 h: *F*_1, 6_ = 7.5, *P* = 0.034; 8 h: *F*_1, 6_ = 6.19, *P* = 0.047), while at 48 h there was a trend for down-regulation of this gene (one-way ANOVA, *F*_1, 4_ = 6.65, *P* = 0.061, Fig. 1H). By contrast, SA and ethephon treatments overall significantly down-regulated *BrLEA4* expression compared to their respective controls (treatment effect, *F*_1, 23_ = 21.68, *P* < 0.001 and *F*_1, 24_ = 43.49, *P* < 0.001, respectively, Table 3, Fig. 1H). The effect of SA treatment on *BrLEA4* expression was time-dependent (interaction effect, *F*_3, 23_ = 9.5, *P* < 0.001, Table 3, Fig. 1H), showing a significant down-regulation at 24 and 48 h after the treatment (Tukey *post hoc* test, 24 h: *P* = 0.006; 48 h: *P* < 0.001, Fig. 1H). The effect of MeJA application on *BrLEA4* expression was time-dependent as well (interaction effect, *F*_3, 23_ = 3.24, *P* = 0.04, Table 3, Fig. 1H). *BrLEA4* expression was significantly down-regulated by MeJA application at 48 h (one-way ANOVA, *F*_1, 5_ = 18.15, *P* = 0.008).

### Effect of hormonal application on phytohormone levels in *B. rapa* shoots and roots

**Figure 2.**
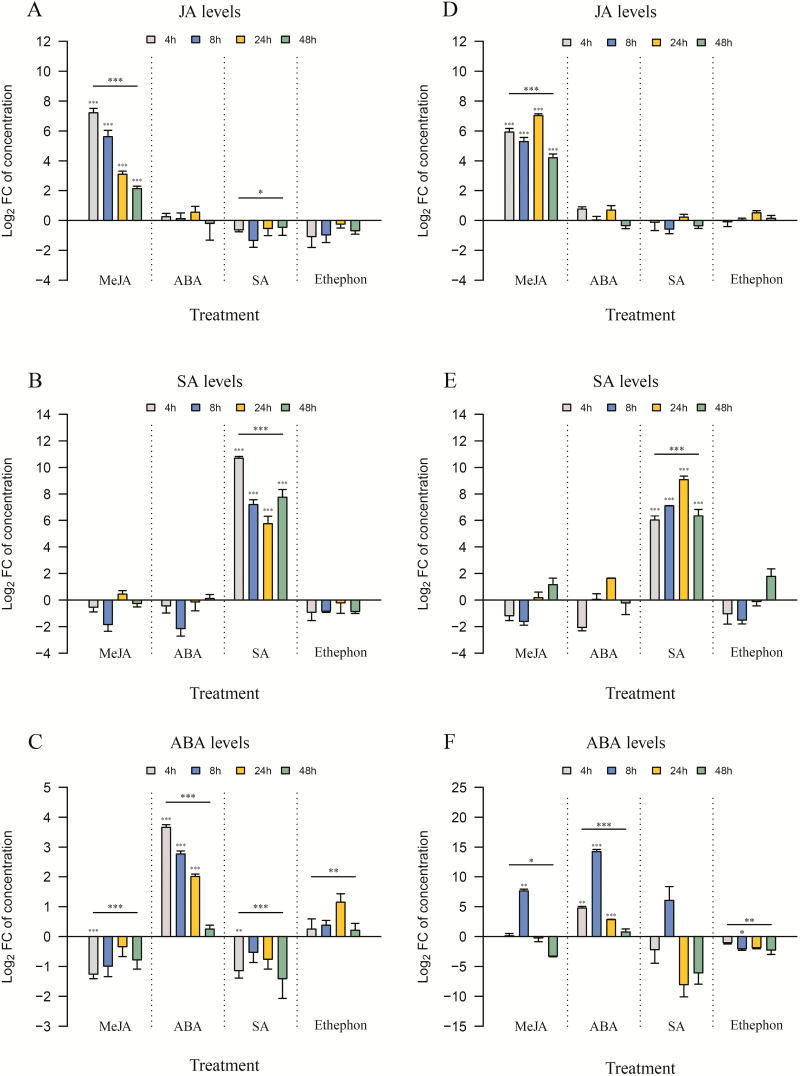
Phytohormone levels in *Brassica rapa* shoots (left column, panels A–C) and roots (right column, panels D–F) in response to hormonal application. The levels of (A, D) JA, (B, E) SA and (C, F) ABA were measured at 4, 8, 24 and 48 h after local MeJA, ABA, SA or ethephon application. Bars represent log_2_ fold changes (log_2_ FC + SE) of concentrations in relation to the respective control. In each hormonal treatment, asterisks over the horizontal line represent the overall significant treatment main effect and those over individual bars indicate significant differences between the treatment group and their respective control plants, according to two-way ANOVA (*n* = 3–4 per treatment and harvest time, except for ABA-treated roots at 24 h where *n* = 2) *P ≤ 0.05; **P ≤ 0.01; ***P ≤ 0.001.

In *B. rapa* shoots, JA levels were significantly increased by MeJA application compared to control plants (treatment effect, *F*_1, 19_ = 485.13, *P* < 0.001, Table 4, Fig. 2A). Salicylic acid application, on the other hand, significantly reduced JA levels (treatment effect, *F*_1, 22_ = 5.81, *P* = 0.025), while neither ABA nor ethephon application had significant effects on JA levels in shoots (treatment effect, *F*_1, 22_ = 0.57, *P* = 0.458 and *F*_1, 24_ = 2.23, *P* = 0.148, respectively, Table 4, Fig. 2A). In *B. rapa* roots, a similar pattern emerged: MeJA application significantly increased JA levels, while ABA, SA or ethephon application did not have any effect on root JA levels when compared to the respective control plants (treatment effect, *F*_1, 22_ = 1240.19, *P* < 0.001; *F*_1, 21_ = 2.34, *P* = 0.141; *F*_1, 23_ = 0.89, *P* = 0.355 and *F*_1, 23_ = 2.08, *P* = 0.163, respectively, Table 5, Fig. 2D). Shoot SA levels were significantly increased by SA treatment (treatment effect, *F*_1, 19_ = 912.11, *P* < 0.001, Table 4, Fig. 2B). Methyl jasmonate, ABA or ethephon application had no significant effect on SA shoot levels (treatment effect, *F*_1, 22_ = 1.96, *P* = 0.175; *F*_1, 22_ = 1.93, *P* = 0.179 and *F*_1, 24_ = 0.03, *P* = 0.873, respectively, Table 4, Fig. 2B). In roots, SA levels were also significantly increased in response to SA application, while MeJA, ABA or ethephon application had no significant effect on root SA levels (treatment effect, *F*_1, 23_ = 677.76, *P* < 0.001; *F*_1, 22_ = 0.33, *P* = 0.573; *F*_1, 21_ = 0.18, *P* = 0.679 and *F*_1, 23_ = 0.07, *P* = 0.793, respectively, Table 5, Fig. 2E).

**Table 4.** Effects of local hormonal application on phytohormone levels in *Brassica rapa* shoots. The levels of JA, SA and ABA were measured at 4, 8, 24 and 48 h after MeJA, ABA, SA and ethephon application. For each measured phytohormone, the data were analysed per treatment using a two-way ANOVA model containing treatment (control, hormonal application), time (4, 8, 24, 48 h) and their interaction term as factors (*n* = 3–4 per treatment and harvest time). Statistically significant effects (*P* ≤ 0.05) are indicated in bold.

Treatment	Factor	Measured phytohormone		
		JA	SA	ABA
MeJA	Treatment (1)	***F***_**1, 19**_ **= 485.13, *P* < 0.001**	*F*_1, 22_ = 1.96, *P* = 0.175	***F***_**1, 21**_ **= 22.82, *P* < 0.001**
	Time (2)	***F***_**3, 19**_ **= 68.46, *P* < 0.001**	***F***_**3, 22**_ **= 17.41, *P* < 0.001**	***F***_**3, 21**_ **= 7.67, *P* = 0.001**
	Interaction (1.2)	***F***_**3, 19**_ **= 35.80, *P* < 0.001**	***F***_**3, 22**_ **= 4.00, *P* = 0.02**	*F*_3, 21_ = 1.22, *P* = 0.327
ABA	Treatment (1)	*F*_1, 22_ = 0.57, *P* = 0.458	*F*_1, 22_ = 1.93, *P* = 0.179	***F***_**1, 20**_ **= 619.21, *P* < 0.001**
	Time (2)	*F*_3, 22_ = 2.69, *P* = 0.071	***F***_**3, 22**_ **= 7.87, *P* = 0.001**	***F***_**3, 20**_ **= 80.47, *P* < 0.001**
	Interaction (1.2)	*F*_3, 22_ = 0.19, *P* = 0.902	*F*_3, 22_ = 2.58, *P* = 0.079	***F***_**3, 20**_ **= 75.15, *P* < 0.001**
SA	Treatment (1)	***F***_**1, 22**_ **= 5.81, *P* = 0.025**	***F***_**1, 19**_ **= 912.11, *P* < 0.001**	***F***_**1, 21**_ **= 15.09, *P* = 0.001**
	Time (2)	*F*_3, 22_ = 2.22, *P* = 0.114	***F***_**3, 19**_ **= 39.42, *P* < 0.001**	***F***_**3, 21**_ **= 3.7, *P* = 0.028**
	Interaction (1.2)	*F*_3, 22_ = 0.29, *P* = 0.834	***F***_**3, 19**_ **= 13.89, *P* < 0.001**	*F*_3, 21_ = 0.6, *P* = 0.62
Ethephon	Treatment (1)	*F*_1, 24_ = 2.23, *P* = 0.148	*F*_1, 24_ = 0.03, *P* = 0.873	***F***_**1, 24**_ **= 10.93, *P* = 0.003**
	Time (2)	*F*_3, 24_ = 1.22, *P* = 0.323	*F*_3, 24_ = 1.14, *P* = 0.353	*F*_3, 24_ = 2.18, *P* = 0.117
	Interaction (1.2)	*F*_3, 24_ = 0.34, *P* = 0.794	*F*_3, 24_ = 0.34, *P* = 0.798	*F*_3, 24_ = 1.94, *P* = 0.149

**Table 5.** Effects of local hormonal application on phytohormone levels in *Brassica rapa* roots. The levels of JA, SA and ABA were measured at 4, 8, 24 and 48 h after MeJA, ABA, SA and ethephon application. For each measured phytohormone, the data were analysed per treatment using a two-way ANOVA model containing treatment (control, hormonal application), time (4, 8, 24, 48 h) and their interaction term as factors (*n* = 3–4 per treatment and harvest time, except for ABA treatment at 24 h where *n* = 2). Statistically significant effects (*P* ≤ 0.05) are indicated in bold.

Treatment	Factor	Measured phytohormone		
		JA	SA	ABA
MeJA	Treatment (1)	***F***_**1, 22**_ **= 1240.19, *P* < 0.001**	*F*_1, 22_ = 0.33, *P* = 0.573	***F***_**1, 18**_ **= 7.07, *P* = 0.016**
	Time (2)	***F***_**3, 22**_ **= 8.59, *P* < 0.001**	***F***_**3, 22**_ **= 3.07, *P* = 0.049**	***F***_**3, 18**_ **= 16.12, *P* < 0.001**
	Interaction (1.2)	***F***_**3, 22**_ **= 14.17, *P* < 0.001**	***F***_**3, 22**_ **= 4.05, *P* = 0.02**	***F***_**3, 18**_ **= 9.83, *P* < 0.001**
ABA	Treatment (1)	*F*_1, 21_ = 2.34, *P* = 0.14	*F*_1, 21_ = 0.18, *P* = 0.679	***F***_**1, 19**_ **= 98.24, *P* < 0.001**
	Time (2)	*F*_3, 21_ = 1.69, *P* = 0.199	*F*_3, 21_ = 2.98, *P* = 0.055	***F***_**3, 19**_ **= 7.69, *P* = 0.001**
	Interaction (1.2)	*F*_3, 21_ = 2.77, *P* = 0.067	***F***_**3, 21**_ **= 3.11, *P* = 0.048**	***F***_**3, 19**_ **= 20.21, *P* < 0.001**
SA	Treatment (1)	*F*_1, 23_ = 0.89, *P* = 0.355	***F***_**1, 23**_ **= 677.76, *P* < 0.001**	*F*_1, 21_ = 2.22, *P* = 0.151
	Time (2)	***F***_**3, 23**_ **= 3.48, *P* = 0.032**	***F***_**3, 23**_ **= 9.88, *P* < 0.001**	*F*_3, 21_ = 2.76, *P* = 0.068
	Interaction (1.2)	*F*_3, 23_ = 0.67, *P* = 0.581	***F***_**3, 23**_ **= 4.90, *P* = 0.009**	***F***_**3, 21**_ **= 4.44, *P* = 0.015**
Ethephon	Treatment (1)	*F*_1, 23_ = 2.08, *P* = 0.163	*F*_1, 23_ = 0.07, *P* = 0.793	***F***_**1, 21**_ **= 10.21, *P* = 0.004**
	Time (2)	*F*_3, 23_ = 1.77, *P* = 0.18	***F***_**3, 23**_ **= 3.08, *P* = 0.048**	***F***_**3, 21**_ **= 3.02, *P* = 0.053**
	Interaction (1.2)	*F*_3, 23_ = 0.47, *P* = 0.71	*F*_3, 23_ = 2.64, *P* = 0.074	*F*_3, 21_ = 0.14, *P* = 0.935

Shoot ABA levels were significantly increased in response to both ABA or ethephon application (treatment effect, *F*_1, 20_ = 619.21, *P* < 0.001 and *F*_1, 24_ = 10.93, *P* = 0.003, respectively, Table 4, Fig. 2C). The effect of ABA application on ABA levels was time-dependent (interaction effect, *F*_3, 20_ = 75.15, *P* < 0.001, Table 4), with statistically significant increases at 4, 8 and 24 h after treatment compared to the respective control plants (Tukey *post hoc* test, *P* < 0.001 for each time point, Fig. 2C). Methyl jasmonate and SA shoot application significantly reduced ABA levels (treatment effect, *F*_1, 21_ = 22.82, *P* < 0.001 and *F*_1, 21_ = 15.09, *P* = 0.001, respectively, Table 4, Fig. 2C). In roots, as expected, ABA levels were significantly increased in response to ABA application, but in contrast to shoots also after MeJA application (treatment effect, *F*_1, 19_ = 98.24, *P* < 0.001 and *F*_1, 18_ = 7.07, *P* = 0.016, respectively, Table 5, Fig. 2F). The effect of both ABA and MeJA application on ABA levels was time-dependent (interaction effect, *F*_3, 19_ = 20.21, *P* < 0.001 and *F*_3, 18_ = 9.83, *P* < 0.001, respectively, Table 5, Fig. 2F). Abscisic acid-treated roots showed significant increases in ABA levels at 4, 8 and 24 h after the treatment (Tukey *post hoc* test, *P* = 0.013, *P* < 0.001 and *P* = 0.034, respectively, Fig. 2F), whereas MeJA application increased ABA levels at 8 h after the treatment compared to control plants (Tukey *post hoc* test, *P* = 0.003, Fig. 2F). Salicylic acid root application had no significant effect on ABA levels, whereas ethephon application significantly reduced ABA levels compared to the respective control plants (treatment effect, *F*_1, 21_ = 2.22, *P* = 0.151 and *F*_1, 21_ = 10.21, *P* = 0.004, respectively, Table 5, Fig. 2F).

## Discussion

**Figure 3.**
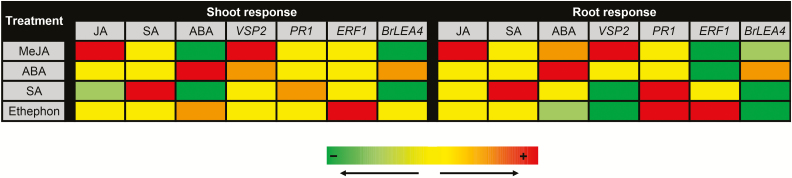
Summarizing scheme of the changes in the phytohormone levels and hormonal-related marker genes in *Brassica rapa* shoots and roots after local hormonal elicitation. Light green indicates reduction/down-regulation and dark green indicates a strong reduction/down-regulation of phytohormone/gene expression levels measured in the same treated organ. Orange indicates increase/up-regulation of phytohormone/gene expression levels and a strong increase/up-regulation is indicated in red. Yellow indicates no changes compared to the respective control group.

Over recent years, many experimental studies have greatly improved our understanding of hormonal-mediated regulation of plant immunity. These studies also identified marker genes for the main defence-related hormonal pathways. However, most of the knowledge on plant immunity and molecular markers is restricted to shoot organs, and in a great extent to the *Arabidopsis* model. In this study, we found that while the majority of the widely used marker genes are regulated by hormonal elicitation in *B. rapa* similarly to in *Arabidopsis*, some of the markers are not unique to a single pathway (Fig. 3). Moreover, our data demonstrate that the response of the marker genes to certain hormonal pathways further differs between roots and shoots.

In line with previous studies in *Arabidopsis* we found that *VSP2* was up-regulated in shoots by local MeJA elicitation. This is showing that *VSP2* is a marker gene of the ABA-regulated MYC-branch of the JA pathway (Anderson *et al.* 2004; Lorenzo *et al.* 2004; Verhage *et al.* 2011; Vos *et al.* 2013). Further studies using *Brassica* species revealed that *VSP2* is also up-regulated by elicitation with JA or MeJA in the shoots (Abe *et al.* 2011; Mathur *et al.* 2013; Tytgat *et al.* 2013; Lee and Hong 2015), indicating that *VSP2* responsiveness to JA is conserved among different plant species of the Brassicaceae. Interestingly, we found that ABA shoot application similarly induced the up-regulation of *VSP2* without affecting JA accumulation, indicating that in *B. rapa*, *VSP2* expression can be elicited independently of JA accumulation in the shoot. Consequently, studies investigating the mechanisms underlying herbivore- or pathogen-induced responses in *Brassica* shoots should consider that *VSP2* up-regulation can be associated with the activation of not only JA-dependent but also of ABA-dependent defences, whereby the latter may be independent of JA accumulation.

In contrast to shoot analyses, those of roots showed that MeJA application resulted in both JA and ABA accumulation and lead to *VSP2* up-regulation. On the other hand, elicitation with ABA and accumulation of ABA alone (without increasing JA levels) did not affect *VSP2* regulation. Taken together, these results indicate that *VSP2* is a more suitable and exclusive marker gene for the JA pathway in *B. rapa* roots than in shoots. Our findings were unexpected as a previous study showed that *VSP2* expression was not affected in *B. oleracea* roots at 6, 18 or 30 h after JA application (Tytgat *et al.* 2013). The differences between our findings and those of Tytgat *et al.* (2013) are probably due to differences in phytohormone concentrations and mode of application. Interestingly, we found a temporal delay in the induction of *VSP2* in roots compared to that in shoots, even though JA levels increased similarly within 4 h in both organs. This raises the question whether roots and shoots process increases in JA levels differently, e.g. by making different variants of the many possible conjugates (Erb and Glauser 2010).

Expression analysis of *PR1* in *B. rapa* shoots confirmed that this gene is a unique marker gene for the SA pathway. It was induced specifically after activation of the SA pathway and not by the JA, ABA or ET pathways. Our results are in line with studies in *Arabidopsis* and *Brassica* spp. shoots showing that *PR1* is a suitable marker gene for the SA pathway (Zhang *et al.* 2003; Nobuta *et al.* 2007; Abe *et al.* 2011; Lee and Hong 2015). Moreover, *PR1* is also a widely used marker for the SA pathway in tomato, maize or soybean, among other species (Martínez-Medina *et al.* 2013; Fernández *et al.* 2014). Surprisingly, and in contrast to shoot behaviour, our results showed that in *B. rapa* roots *PR1* was induced not only in response to SA treatment, but also after ethephon application even though the latter did not affect SA levels. These findings indicate that *PR1* induction in the roots does not necessarily require SA accumulation, supporting the notion that shoots and roots respond differently to specific hormonal pathways. Studies on the interactions between plants and soil biota often assess changes in the *PR1* expression as a marker for the activation of the SA pathway, following the paradigms based on shoot-derived data (Paparu *et al.* 2007; Chen *et al.* 2016; Martínez‐Medina *et al.* 2017a, b). Although ethephon could have an effect that is independent of ET (Lawton *et al.* 1994), our results suggest that specifically in *B. rapa*, *PR1* may also respond to ET elicitation.

Our data also demonstrated that *ERF1* is a suitable marker gene for the ET pathway in *B. rapa* shoots as well as in roots. We found *ERF1* up-regulation specifically in ethephon-treated shoots, where surprisingly, ABA levels were also increased. However, ABA accumulation alone was not sufficient to induce *ERF1* expression, as the expression of this gene was not affected in ABA-treated shoots. *ERF1* is a widely used marker gene for ET pathway in different plant species, such as tomato, legumes and *Arabidopsis* (Anderson *et al.* 2010; Tian *et al.* 2014; Broekgaarden *et al.* 2015; Huang *et al.* 2015). Studies on *Arabidopsis* shoots have shown that *ERF1* can be activated by the ET or the JA pathway, or synergistically by both phytohormones. Jasmonic acid-induced *ERF1* expression is associated with activation of the ERF-branch of the JA pathway, which is co-regulated by ET (Penninckx *et al.* 1998; Solano *et al.* 1998; Lorenzo *et al.* 2003; Robert-Seilaniantz *et al.* 2011; Pieterse *et al.* 2012). In *Arabidopsis*, ABA is known to act antagonistically on the ERF-branch of the JA pathway (Lorenzo *et al.* 2004; Verhage *et al.* 2011; Vos *et al.* 2013). However, the fact that we found *ERF1* up-regulation together with increased ABA levels in ethephon-treated shoots suggests that ABA might not have an antagonistic effect on the ERF-branch of the JA pathway in *B. rapa* shoots, as it does in *Arabidopsis* shoots. In contrast, ethephon application to roots decreased ABA levels and lead to *ERF1* up-regulation, indicating that ABA and ET indeed may act antagonistically on *ERF1* expression in *B. rapa* roots.

We further tested *BrLEA4* as a novel marker gene for the ABA pathway in *B. rapa*. We showed that *BrLEA4* is an early ABA-inducible gene in *B. rapa* shoots and roots. *LEA* genes, encoding for late-embryogenesis abundant proteins, are used as molecular markers for the ABA pathway in different plant species, such as soybean, tomato, maize and other (Martínez-Medina *et al.* 2013; Fernández *et al.* 2014; Zamora-Briseño and de Jiménez 2016). Among different *LEA* genes, *LEA4* was shown to be highly ABA inducible in vegetative tissues of *Arabidopsis* and *Brassica* spp. (Hoth *et al.* 2002; Dalal *et al.* 2009). Remarkably, we found that in both shoots and roots, JA or SA accumulation down-regulated *BrLEA4* expression. In the shoots this coincided with suppressed ABA levels, further suggesting that ABA accumulation is required for *BrLEA4* induction in shoots. Interestingly, ethephon application to the roots, which suppressed local ABA levels, also resulted in *BrLEA4* down-regulation. Taken together, our findings suggest that *BrLEA4* is a suitable marker gene for ABA pathway in *B. rapa* shoots and roots. In addition, our results showed that elicitation of the JA or the SA pathway in shoots and the JA, SA or ET pathways in roots suppresses the induction of this gene in *B. rapa*, further supporting the notion that *BrLEA4* is a unique marker gene for the ABA pathway. Based on our results we therefore suggest *VSP2* as a marker gene for the JA or ABA pathways, *PR1* as a marker gene for the SA pathway, *ERF1* for the ET pathway and *BrLEA4* for the ABA pathway in *B. rapa* shoots. For *B. rapa* roots, we propose *VSP2* as a marker gene for the JA pathway, *PR1* for the SA or ET pathways, *ERF1* for the ET pathway and *BrLEA4* for the ABA pathway.

In addition to the effects of single hormonal applications on specific marker gene responses, we also found evidence for hormonal crosstalk. For example, SA application reduced JA levels, especially in shoots. This may have been the reason that *VSP2* was down-regulated in shoots and roots of SA-treated *B. rapa* (Koornneef *et al.* 2008; Pieterse *et al.* 2009). Methyl jasmonate application, on the other hand, only mildly repressed SA or *PR1* expression. Jasmonic acid–salicylic acid negative crosstalk is one of the best-described hormonal interaction processes (Pieterse *et al.* 2009). However, the nature of the interaction is highly dynamic and may also involve ET (Koornneef *et al.* 2008; Leon-Reyes *et al.* 2010). The negative crosstalk between ABA and ET is also well described, especially in the context of regulating abiotic stress responses. Increases in ABA levels lead to stomatal closure, which is a functional adaptation to drought stress (Nguyen *et al.* 2016). Ethylene, on the other hand, is involved in responses to flooding and waterlogging. Its production may result in the formation of adventitious root aerenchyma to overcome anoxia or quiescence responses that help the plant to survive while (partly) under water. In response to complete flooding, ET may also stimulate shoot elongation to ensure shoot contact with the air before asphyxiating (Pierik *et al.* 2006). We found evidence for negative ET–ABA crosstalk in the roots of *B. rapa* since ethephon treatment reduced ABA levels and increased *ERF1* expression. Interestingly, in the shoots we observed that ethephon treatment enhances ABA levels. Despite this increase in shoot ABA, *ERF1* expression was increased by ethephon application as well, but not as strongly as in the roots. Possibly these differences between root and shoot hormonal responses are related to differences in the interactions they experience in their natural environment. In general, hormonal crosstalk is very dynamic and complex. Future studies, for example with combined applications of multiple phytohormones (Koornneef *et al.* 2008), could shed more light on the nature of hormonal crosstalk processes in *B. rapa*.

## Conclusions

Although most of the *Arabidopsis*-derived marker genes tested in this study are also suitable markers in *B. rapa*, some e.g., *VSP2* and *PR1* fail to show specificity for one pathway. Furthermore, we demonstrated that the responsiveness of some marker genes to specific phytohormones is organ specific since roots behaved differently to shoots. Consequently, plant organ should be taken into consideration in marker gene selection. Overall, our findings suggest that the link between marker gene expression profiles and the activation of specific hormone-inducible pathways should be interpreted with caution. It is therefore advisable to combine analyses of multiple marker genes with those of phytohormone levels to ascertain more certainly which hormonally regulated defence pathways are activated.

## Sources of Funding

This research was supported by the German Centre for Integrative Biodiversity Research (iDiv) Halle-Jena-Leipzig funded by the German Research Foundation (FZT 118). A.M.-M. further acknowledges the support by the programme to support junior researchers to obtain third-party funding from Friedrich-Schiller-Universität Jena (DRM/2015-02). The authors acknowledge support from the iDiv Open Science Publication Fund.

## Contributions by the Authors

G.V.P., A.M.-M. and N.M.V.D. designed the research; G.V.P. performed the experiments; G.V.P. and A.M. performed qRT–PCR analysis; G.V.P. and K.G. analysed phytohormone levels; G.V.P. performed the statistical analyses; G.V.P., A.M.-M. and N.M.V.D. executed data interpretation and wrote the manuscript with input from all the authors.

## Conflict of Interest

None declared.

## Supporting Information

The following additional information is available in the online version of this article—

**Figure S1**. Amplification efficiencies of the primers used in this study.

**Table S1**. Relative expression (SE) of marker genes in *Brassica rapa* shoots in response to hormonal application to the shoots.

**Table S2**. Relative expression (SE) of marker genes in *Brassica rapa* roots in response to hormonal application to the roots.

**Table S3**. Levels of phytohormones (SE) in *Brassica rapa* shoots and roots in response to hormonal application.

**Table S4**. *F*- and *P*-values of a two-way ANOVA model on gene expression levels in *Brassica rapa* shoots and roots.

**Table S5**. *F*- and *P*-values of a two-way ANOVA model on the phytohormone levels in *Brassica rapa* shoots and roots.

Supplementary Material

## References

[CIT0001] Abe H, Narusaka Y, Sasaki I, Hatakeyama K, Shin-I S, Narusaka M, Fukami-Kobayashi K, Matsumoto S, Kobayashi M 2011 Development of full-length cDNAs from Chinese cabbage (*Brassica rapa* subsp. *pekinensis*) and identification of marker genes for defence response. DNA Research 18:277–289. 21745830 10.1093/dnares/dsr018 PMC3158467

[CIT0002] Anderson JP, Badruzsaufari E, Schenk PM, Manners JM, Desmond OJ, Ehlert C, Maclean DJ, Ebert PR, Kazan K 2004 Antagonistic interaction between abscisic acid and jasmonate-ethylene signaling pathways modulates defense gene expression and disease resistance in *Arabidopsis*. The Plant Cell 16:3460–3479. 15548743 10.1105/tpc.104.025833 PMC535886

[CIT0003] Anderson JP, Lichtenzveig J, Gleason C, Oliver RP, Singh KB 2010 The B-3 ethylene response factor MtERF1-1 mediates resistance to a subset of root pathogens in *Medicago truncatula* without adversely affecting symbiosis with rhizobia. Plant Physiology 154:861–873. 20713618 10.1104/pp.110.163949 PMC2949043

[CIT0004] Barr KL, Hearne LB, Briesacher S, Clark TL, Davis GE 2010 Microbial symbionts in insects influence down-regulation of defense genes in maize. PLoS One 5:e11339. 20596533 10.1371/journal.pone.0011339 PMC2893166

[CIT0005] Berrocal‐Lobo M, Molina A, Solano R 2002 Constitutive expression of ETHYLENE‐RESPONSE‐FACTOR1 in *Arabidopsis* confers resistance to several necrotrophic fungi. The Plant Journal 29:23–32. 12060224 10.1046/j.1365-313x.2002.01191.x

[CIT0006] Broekgaarden C, Caarls L, Vos IA, Pieterse CM, Van Wees SC 2015 Ethylene: traffic controller on hormonal crossroads to defense. Plant Physiology 169:2371–2379. 26482888 10.1104/pp.15.01020 PMC4677896

[CIT0007] Chandna R, Augustine R, Bisht NC 2012 Evaluation of candidate reference genes for gene expression normalization in *Brassica juncea* using real time quantitative RT-PCR. PLoS One 7:e36918. 22606308 10.1371/journal.pone.0036918 PMC3350508

[CIT0008] Chen T, Bi K, He Z, Gao Z, Zhao Y, Fu Y, Cheng J, Xie J, Jiang D 2016 *Arabidopsis* mutant bik1 exhibits strong resistance to *Plasmodiophora brassicae*. Frontiers in Physiology 7:402. 27679580 10.3389/fphys.2016.00402 PMC5020103

[CIT0009] Chen X, Truksa M, Shah S, Weselake RJ 2010 A survey of quantitative real-time polymerase chain reaction internal reference genes for expression studies in *Brassica napus*. Analytical Biochemistry 405:138–140. 20522329 10.1016/j.ab.2010.05.032

[CIT0010] Dalal M, Tayal D, Chinnusamy V, Bansal KC 2009 Abiotic stress and ABA-inducible group 4 LEA from *Brassica napus* plays a key role in salt and drought tolerance. Journal of Biotechnology 139:137–145. 19014980 10.1016/j.jbiotec.2008.09.014

[CIT0011] Dombrecht B, Xue GP, Sprague SJ, Kirkegaard JA, Ross JJ, Reid JB, Fitt GP, Sewelam N, Schenk PM, Manners JM, Kazan K 2007 MYC2 differentially modulates diverse jasmonate-dependent functions in *Arabidopsis*. The Plant Cell 19:2225–2245. 17616737 10.1105/tpc.106.048017 PMC1955694

[CIT0012] Erb M, Glauser G 2010 Family business: multiple members of major phytohormone classes orchestrate plant stress responses. Chemistry-A European Journal 16:10280–10289. 10.1002/chem.201001219 20648494

[CIT0013] Fernández I, Merlos M, López-Ráez JA, Martínez-Medina A, Ferrol N, Azcón C, Bonfante P, Flors V, Pozo MJ 2014 Defense related phytohormones regulation in arbuscular mycorrhizal symbioses depends on the partner genotypes. Journal of Chemical Ecology 40:791–803. 24997625 10.1007/s10886-014-0473-6

[CIT0014] Gaffney T, Friedrich L, Vernooij B, Negrotto D, Nye G, Uknes S, Ward E, Kessmann H, Ryals J 1993 Requirement of salicylic acid for the induction of systemic acquired resistance. Science 261:754–756. 17757215 10.1126/science.261.5122.754

[CIT0015] Hoth S, Morgante M, Sanchez JP, Hanafey MK, Tingey SV, Chua NH 2002 Genome-wide gene expression profiling in *Arabidopsis thaliana* reveals new targets of abscisic acid and largely impaired gene regulation in the abi1-1 mutant. Journal of Cell Science 115:4891–4900. 12432076 10.1242/jcs.00175

[CIT0016] Huang PY, Catinot J, Zimmerli L 2015 Ethylene response factors in *Arabidopsis* immunity. Journal of Experimental Botany 67:1231–1241. 26663391 10.1093/jxb/erv518

[CIT0017] Hundertmark M, Hincha DK 2008 LEA (late embryogenesis abundant) proteins and their encoding genes in *Arabidopsis thaliana*. BMC Genomics 9:118. 18318901 10.1186/1471-2164-9-118 PMC2292704

[CIT0018] Kazan K, Manners JM 2013 MYC2: the master in action. Molecular Plant 6:686–703. 23142764 10.1093/mp/sss128

[CIT0019] Koornneef A, Leon-Reyes A, Ritsema T, Verhage A, Den Otter FC, Van Loon LC, Pieterse CM 2008 Kinetics of salicylate-mediated suppression of jasmonate signaling reveal a role for redox modulation. Plant Physiology 147:1358–1368. 18539774 10.1104/pp.108.121392 PMC2442557

[CIT0020] Kroes A, Stam JM, David A, Boland W, van Loon JJ, Dicke M, Poelman EH 2016 Plant-mediated interactions between two herbivores differentially affect a subsequently arriving third herbivore in populations of wild cabbage. Plant Biology 18:981–991. 27492059 10.1111/plb.12490

[CIT0021] Lawrence SD, Novak NG, Kayal WE, Ju CJ, Cooke JE 2012 Root herbivory: molecular analysis of the maize transcriptome upon infestation by Southern corn rootworm, *Diabrotica undecimpunctata howardi*. Physiologia Plantarum 144:303–319. 22172013 10.1111/j.1399-3054.2011.01557.x

[CIT0022] Lawton KA, Potter SL, Uknes S, Ryals J 1994 Acquired resistance signal transduction in *Arabidopsis* is ethylene independent. The Plant Cell 6:581–588. 12244251 10.1105/tpc.6.5.581 PMC160460

[CIT0023] Lee Y, Hong J 2015 Differential defence responses of susceptible and resistant kimchi cabbage cultivars to anthracnose, black spot and black rot diseases. Plant Pathology 64:406–415.

[CIT0024] Leon-Reyes A, Du Y, Koornneef A, Proietti S, Körbes AP, Memelink J, Pieterse CM, Ritsema T 2010 Ethylene signaling renders the jasmonate response of *Arabidopsis* insensitive to future suppression by salicylic acid. Molecular Plant-Microbe Interactions 23:187–197. 20064062 10.1094/MPMI-23-2-0187

[CIT0025] Leon-Reyes A, Spoel SH, De Lange ES, Abe H, Kobayashi M, Tsuda S, Millenaar FF, Welschen RA, Ritsema T, Pieterse CM 2009 Ethylene modulates the role of NONEXPRESSOR OF PATHOGENESIS-RELATED GENES1 in cross talk between salicylate and jasmonate signaling. Plant Physiology 149: 1797–1809. 19176718 10.1104/pp.108.133926 PMC2663751

[CIT0026] Livak KJ, Schmittgen TD 2001 Analysis of relative gene expression data using real-time quantitative PCR and the 2(-delta delta C(T)) method. Methods 25:402–408. 11846609 10.1006/meth.2001.1262

[CIT0027] Lorenzo O, Chico JM, Sánchez-Serrano JJ, Solano R 2004 JASMONATE-INSENSITIVE1 encodes a MYC transcription factor essential to discriminate between different jasmonate-regulated defense responses in *Arabidopsis*. The Plant Cell 16:1938–1950. 15208388 10.1105/tpc.022319 PMC514172

[CIT0028] Lorenzo O, Piqueras R, Sánchez-Serrano JJ, Solano R 2003 ETHYLENE RESPONSE FACTOR1 integrates signals from ethylene and jasmonate pathways in plant defense. The Plant Cell 15:165–178. 12509529 10.1105/tpc.007468 PMC143489

[CIT0029] Lorenzo O, Solano R 2005 Molecular players regulating the jasmonate signalling network. Current Opinion in Plant Biology 8:532–540. 16039901 10.1016/j.pbi.2005.07.003

[CIT0030] Lu J, Robert CA, Riemann M, Cosme M, Mène-Saffrané L, Massana J, Stout MJ, Lou Y, Gershenzon J, Erb M 2015 Induced jasmonate signaling leads to contrasting effects on root damage and herbivore performance. Plant Physiology 167:1100–1116. 25627217 10.1104/pp.114.252700 PMC4348761

[CIT0031] Maag D, Kandula DR, Müller C, Mendoza-Mendoza A, Wratten SD, Stewart A, Rostás M 2014 Trichoderma atroviride LU132 promotes plant growth but not induced systemic resistance to *Plutella xylostella* in oilseed rape. BioControl 59:241–252.

[CIT0032] Machado RA, Ferrieri AP, Robert CA, Glauser G, Kallenbach M, Baldwin IT, Erb M 2013 Leaf-herbivore attack reduces carbon reserves and regrowth from the roots via jasmonate and auxin signaling. The New Phytologist 200:1234–1246. 23914830 10.1111/nph.12438

[CIT0033] Martínez-Medina A, Appels FV, Van Wees SC 2017a Impact of salicylic acid-and jasmonic acid-regulated defences on root colonization by *Trichoderma harzianum* T-78. Plant Signaling & Behavior 12:e1345404. 28692334 10.1080/15592324.2017.1345404 PMC5616143

[CIT0034] Martínez‐Medina A, Fernandez I, Lok GB, Pozo MJ, Pieterse CM, Van Wees S 2017b Shifting from priming of salicylic acid‐to jasmonic acid‐regulated defences by *Trichoderma* protects tomato against the root knot nematode *Meloidogyne incognita*. New Phytologist 213:1363–1377. 27801946 10.1111/nph.14251

[CIT0035] Martínez-Medina A, Fernández I, Sánchez-Guzmán MJ, Jung SC, Pascual JA, Pozo MJ 2013 Deciphering the hormonal signalling network behind the systemic resistance induced by *Trichoderma harzianum* in tomato. Frontiers in Plant Science 4:206. 23805146 10.3389/fpls.2013.00206 PMC3690380

[CIT0036] Mathur V, Tytgat TO, Hordijk CA, Harhangi HR, Jansen JJ, Reddy AS, Harvey JA, Vet LE, van Dam NM 2013 An ecogenomic analysis of herbivore-induced plant volatiles in *Brassica juncea*. Molecular Ecology 22:6179–6196. 24219759 10.1111/mec.12555

[CIT0037] Nguyen D, D’Agostino N, Tytgat TO, Sun P, Lortzing T, Visser EJ, Cristescu SM, Steppuhn A, Mariani C, Dam NM 2016 Drought and flooding have distinct effects on herbivore‐induced responses and resistance in *Solanum dulcamara*. Plant, Cell & Environment 39:1485–1499. 10.1111/pce.12708 26759219

[CIT0038] Nobuta K, Okrent RA, Stoutemyer M, Rodibaugh N, Kempema L, Wildermuth MC, Innes RW 2007 The GH3 acyl adenylase family member PBS3 regulates salicylic acid-dependent defense responses in *Arabidopsis*. Plant Physiology 144:1144–1156. 17468220 10.1104/pp.107.097691 PMC1914169

[CIT0039] Papadopoulou GV, van Dam NM 2017 Mechanisms and ecological implications of plant-mediated interactions between belowground and aboveground insect herbivores. Ecological Research 32:13–26.

[CIT0040] Paparu P, Dubois T, Coyne D, Viljoen A 2007 Defense-related gene expression in susceptible and tolerant bananas (*Musa* spp.) following inoculation with non-pathogenic *Fusarium oxysporum* endophytes and challenge with *Radopholus similis*. Physiological and Molecular Plant Pathology 71:149–157.

[CIT0041] Penninckx IA, Thomma BP, Buchala A, Métraux JP, Broekaert WF 1998 Concomitant activation of jasmonate and ethylene response pathways is required for induction of a plant defensin gene in *Arabidopsis*. The Plant Cell 10:2103–2113. 9836748 10.1105/tpc.10.12.2103 PMC143966

[CIT0042] Pierik R, Tholen D, Poorter H, Visser EJ, Voesenek LA 2006 The Janus face of ethylene: growth inhibition and stimulation. Trends in Plant Science 11:176–183. 16531097 10.1016/j.tplants.2006.02.006

[CIT0043] Pieterse CM, Leon-Reyes A, Van der Ent S, Van Wees SC 2009 Networking by small-molecule hormones in plant immunity. Nature Chemical Biology 5:308–316. 19377457 10.1038/nchembio.164

[CIT0044] Pieterse CM, Van der Does D, Zamioudis C, Leon-Reyes A, Van Wees SC 2012 Hormonal modulation of plant immunity. Annual Review of Cell and Developmental Biology 28:489–521. 10.1146/annurev-cellbio-092910-154055 22559264

[CIT0045] Pineda A, Soler R, Pastor V, Li Y, Dicke M 2017 Plant‐mediated species networks: the modulating role of herbivore density. Ecological Entomology 42:449–457.

[CIT0046] Pré M, Atallah M, Champion A, De Vos M, Pieterse CM, Memelink J 2008 The AP2/ERF domain transcription factor ORA59 integrates jasmonic acid and ethylene signals in plant defense. Plant Physiology 147:1347–1357. 18467450 10.1104/pp.108.117523 PMC2442530

[CIT0047] Robert-Seilaniantz A, Grant M, Jones JD 2011 Hormone crosstalk in plant disease and defense: more than just jasmonate-salicylate antagonism. Annual Review of Phytopathology 49:317–343. 10.1146/annurev-phyto-073009-114447 21663438

[CIT0048] Schäfer M, Brütting C, Baldwin IT, Kallenbach M 2016 High-throughput quantification of more than 100 primary-and secondary-metabolites, and phytohormones by a single solid-phase extraction based sample preparation with analysis by UHPLC–HESI–MS/MS. Plant Methods 12:1. 27239220 10.1186/s13007-016-0130-x PMC4882772

[CIT0049] Solano R, Stepanova A, Chao Q, Ecker JR 1998 Nuclear events in ethylene signaling: a transcriptional cascade mediated by ETHYLENE-INSENSITIVE3 and ETHYLENE-RESPONSE-FACTOR1. Genes & Development 12:3703–3714. 9851977 10.1101/gad.12.23.3703 PMC317251

[CIT0050] Soler R, Badenes‐Pérez FR, Broekgaarden C, Zheng SJ, David A, Boland W, Dicke M 2012 Plant‐mediated facilitation between a leaf‐feeding and a phloem‐feeding insect in a brassicaceous plant: from insect performance to gene transcription. Functional Ecology 26:156–166.

[CIT0051] Tian D, Peiffer M, De Moraes CM, Felton GW 2014 Roles of ethylene and jasmonic acid in systemic induced defense in tomato (*Solanum lycopersicum*) against *Helicoverpa zea*. Planta 239:577–589. 24271004 10.1007/s00425-013-1997-7

[CIT0052] Tsunoda T, Krosse S, van Dam NM 2017 Root and shoot glucosinolate allocation patterns follow optimal defence allocation theory. Journal of Ecology 105:1256–1266.

[CIT0053] Tytgat TO, Verhoeven KJ, Jansen JJ, Raaijmakers CE, Bakx-Schotman T, McIntyre LM, van der Putten WH, Biere A, van Dam NM 2013 Plants know where it hurts: root and shoot jasmonic acid induction elicit differential responses in *Brassica oleracea*. PLoS One 8:e65502. 23776489 10.1371/journal.pone.0065502 PMC3679124

[CIT0054] van Dam NM, Harvey JA, Wäckers FL, Bezemer TM, van der Putten WH, Vet LE 2003 Interactions between aboveground and belowground induced responses against phytophages. Basic and Applied Ecology 4:63–77.

[CIT0055] van der Putten WH 2003 Plant defense belowground and spatiotemporal processes in natural vegetation. Ecology 84:2269–2280.

[CIT0056] van Loon LC, Rep M, Pieterse CM 2006 Significance of inducible defense-related proteins in infected plants. Annual Review of Phytopathology 44:135–162. 10.1146/annurev.phyto.44.070505.143425 16602946

[CIT0057] van Wees SC, Luijendijk M, Smoorenburg I, van Loon LC, Pieterse CM 1999 Rhizobacteria-mediated induced systemic resistance (ISR) in *Arabidopsis* is not associated with a direct effect on expression of known defense-related genes but stimulates the expression of the jasmonate-inducible gene Atvsp upon challenge. Plant Molecular Biology 41:537–549. 10608663 10.1023/a:1006319216982

[CIT0058] Verhage A, Vlaardingerbroek I, Raaymakers C, Van Dam NM, Dicke M, Van Wees SC, Pieterse CM 2011 Rewiring of the jasmonate signaling pathway in *Arabidopsis* during insect herbivory. Frontiers in Plant Science 2:47. 22645537 10.3389/fpls.2011.00047 PMC3355780

[CIT0059] Vos IA, Moritz L, Pieterse CM, Van Wees SC 2015 Impact of hormonal crosstalk on plant resistance and fitness under multi-attacker conditions. Frontiers in Plant Science 6:639. 26347758 10.3389/fpls.2015.00639 PMC4538242

[CIT0060] Vos IA, Verhage A, Schuurink RC, Watt LG, Pieterse CM, Van Wees SC 2013 Onset of herbivore-induced resistance in systemic tissue primed for jasmonate-dependent defenses is activated by abscisic acid. Frontiers in Plant Science 4:539. 24416038 10.3389/fpls.2013.00539 PMC3874679

[CIT0061] Wang X, Wang H, Wang J, Sun R, Wu J, Liu S, Bai Y, Mun J-H, Bancroft I, Cheng F 2011 The genome of the mesopolyploid crop species *Brassica rapa*. Nature Genetics 43:1035–1039. 21873998 10.1038/ng.919

[CIT0062] Zamora-Briseño JA, de Jiménez ES 2016 A LEA 4 protein up-regulated by ABA is involved in drought response in maize roots. Molecular Biology Reports 43:221–228. 26922182 10.1007/s11033-016-3963-5

[CIT0063] Zarate SI, Kempema LA, Walling LL 2007 Silverleaf whitefly induces salicylic acid defenses and suppresses effectual jasmonic acid defenses. Plant Physiology 143:866–875. 17189328 10.1104/pp.106.090035 PMC1803729

[CIT0064] Zhang Y, Tessaro MJ, Lassner M, Li X 2003 Knockout analysis of *Arabidopsis* transcription factors TGA2, TGA5, and TGA6 reveals their redundant and essential roles in systemic acquired resistance. The Plant Cell 15:2647–2653. 14576289 10.1105/tpc.014894 PMC280568

