# Supplementary material for: Defence signalling marker gene responses to hormonal elicitation differ between roots and shoots
Source: AoB Plants. 2018 May 16;10(3):ply031. doi: 10.1093/aobpla/ply031 (PMC6007416; doi:10.1093/aobpla/ply031)
Supplement: Supplementary Material [file ply031_suppl_supplementary_material.docx]

**
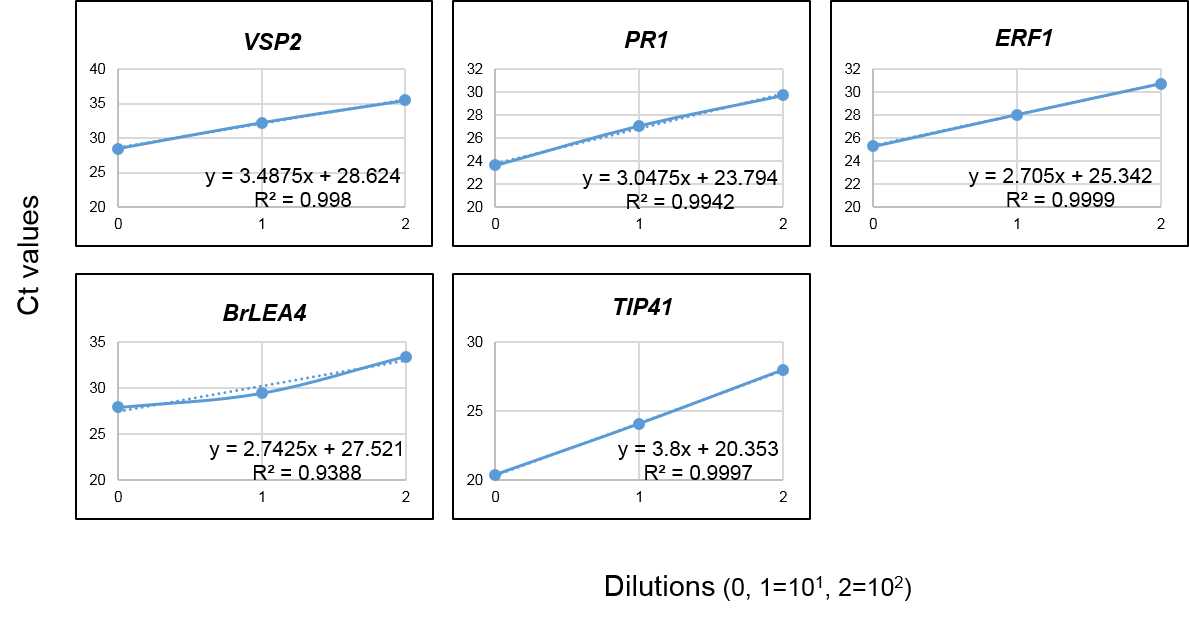
**

**Figure S1** Amplification efficiencies of the primers used in this study

**Table S1.** Relative expression (SE) of marker genes in *Brassica* *rapa* shoots in response to hormonal application to the shoots. The expression levels of *VSP2* (*VEGETATIVE STORAGE PROTEIN 2*), *PR1* (*PATHOGENESIS*-*RELATED* *PROTEIN* *1*), *ERF1* (*ETHYLENE RESPONSE FACTOR 1*) and *BrLEA4* (*Brassica* *rapa* 18 kDa seed maturation protein-like) were measured in the shoots at 4, 8, 24 and 48h after methyl jasmonate (MeJA), abscisic acid (ABA), salicylic acid (SA) or ethephon application. Due to differences in the methodology of ethephon application compared to the other treatments, a different set of plants was used as control for the ethephon treatment. Gene expression was quantified with qRT-PCR and normalized to the *TIP41* housekeeping gene by using the 2^-ΔCT^ method (n = 3-4 per treatment and harvest time)

| **Treatment** | **Time** | ***VSP2*** | |  | ***PR1*** | |  | ***ERF1*** | |  | ***BrLEA4*** |
| --- | --- | --- | --- | --- | --- | --- | --- | --- | --- | --- | --- |
| Control | 4h | 87.38 (53.4) | |  | 3.44 (2.0) | |  | 0.05 (0.02) | |  | 0.23 (0.12) |
|  | 8h | 19.02 (11.9) | |  | 18.26 (8.4) | |  | 0.05 (0.01) | |  | 0.02 (0.01) |
|  | 24h | 13.89 (5.5) | |  | 4.3 (2.5) | |  | 0.08 (0.02) | |  | 0.32 (0.08) |
|  | 48h | 3.64 (2.5) | |  | 1.14 (0.5) | |  | 0.08 (0.02) | |  | 0.64 (0.1) |
|  |  |  | |  |  | |  |  | |  |  |
| MeJA | 4h | 317.88 (188.7) | |  | 1.77 (1.4) | |  | 0.09 (0.02) | |  | 0.09 (0.06) |
|  | 8h | 550.00 (243.7) | |  | 0.75 (0.2) | |  | 0.07 (0.03) | |  | 0.04 (0.02) |
|  | 24h | 94.97 (39.5) | |  | 2.24 (0.8) | |  | 0.06 (0.01) | |  | 0.13 (0.1) |
|  | 48h | 36.76 (25.8) | |  | 5.54 (2.4) | |  | 0.11 (0.03) | |  | 0.1 (0.02) |
|  |  |  | |  |  | |  |  | |  |  |
| ABA | 4h | 31.95 (8.0) | |  | 1.56 (0.5) | |  | 0.07 (0.03) | |  | 2.09 (0.56) |
|  | 8h | 227.81 (163.0) | |  | 9.13 (7.1) | |  | 0.06 (0.01) | |  | 0.03 (0.02) |
|  | 24h | 94.36 (43.9) | |  | 17.79 (10.8) | |  | 0.08 (0.03) | |  | 0.45 (0.22) |
|  | 48h | 37.07 (25.1) | |  | 1.52 (1.0) | |  | 0.08 (0.03) | |  | 0.53 (0.2) |
|  |  |  | |  |  | |  |  | |  |  |
| SA | 4h | 18.35 (11.8) | |  | 65.17 (33.7) | |  | 0.09 (0.07) | |  | 0.06 (0.04) |
|  | 8h | 3.75 (0.9) | |  | 134.04 (33.5) | |  | 0.02 (0.01) | |  | 0.03 (0.01) |
|  | 24h | 2.51 (1.6) | |  | 2.00 (0.3) | |  | 0.1 (0.07) | |  | 0.16 (0.08) |
|  | 48h | 1.49 (1.0) | |  | 7.76 (4.0) | |  | 0.11 (0.05) | |  | 0.13 (0.06) |
|  |  |  |  | |  |  | |  |  | |  |
| Control | 4h | 79.08 (54.8) |  | | 1.78 (0.5) |  | | 0.04 (0.02) |  | | 0.03 (0.01) |
|  | 8h | 4.22 (2.6) |  | | 4.68 (2.2) |  | | 0.02 (0.01) |  | | 0.02 (0.00) |
|  | 24h | 7.37 (4.1) |  | | 6.39 (4.9) |  | | 0.06 (0.02) |  | | 0.08 (0.05) |
|  | 48h | 2.9 (2.3) |  | | 4.51 (2.1) |  | | 0.02 (0.01) |  | | 0.03 (0.02) |
|  |  |  |  | |  |  | |  |  | |  |
|  | 4h | 5.02 (2.3) |  | | 8.18 (6.2) |  | | 0.22 (0.06) |  | | 0.02 (0.01) |
| Ethephon | 8h | 10.23 (6.6) |  | | 6.75 (2.6) |  | | 0.18 (0.08) |  | | 0.02 (0.01) |
|  | 24h | 0.21 (0.1) |  | | 5.72 (2.5) |  | | 0.1 (0.01) |  | | 0.06 (0.03) |
|  | 48h | 3.52 (1.8) |  | | 7.12 (3) |  | | 0.13 (0.05) |  | | 0.15 (0.04) |

**Table S2.** Relative expression (SE) of marker genes in *Brassica* *rapa* roots in response to hormonal application to the roots. The expression levels of *VSP2* (*VEGETATIVE STORAGE PROTEIN 2*), *PR1* (*PATHOGENESIS*-*RELATED* *PROTEIN* *1*), *ERF1* (*ETHYLENE RESPONSE FACTOR 1*) and *BrLEA4* (*Brassica* *rapa* 18 kDa seed maturation protein-like) were measured in the roots at 4, 8, 24 and 48h after methyl jasmonate (MeJA), abscisic acid (ABA), salicylic acid (SA) or ethephon application. Due to differences in the methodology of ethephon application compared to the other treatments, a different set of plants was used as control for the ethephon treatment. Gene expression was quantified with qRT-PCR and normalized to the *TIP41* housekeeping gene by using the 2^-ΔCT^ method (n = 3-4 per treatment and harvest time)

| **Treatment** | **Time** | ***VSP2*** |  | ***PR1*** |  | ***ERF1*** |  | ***BrLEA4*** |
| --- | --- | --- | --- | --- | --- | --- | --- | --- |
| Control | 4h | 0.247 (0.207) |  | 0.043 (0.025) |  | 0.262 (0.087) |  | 0.287 (0.176) |
|  | 8h | 0.07 (0.026) |  | 0.008 (0.006) |  | 0.152 (0.01) |  | 0.033 (0.020) |
|  | 24h | 0.012 (0.009) |  | 0.026 (0.015) |  | 0.066 (0.017) |  | 0.466 (0.25) |
|  | 48h | 0.003 (0.001) |  | 0.021 (0.012) |  | 0.088 (0.019) |  | 4.85 (1.209) |
|  |  |  |  |  |  |  |  |  |
| MeJA | 4h | 0.3 (0.235) |  | 0.039 (0.034) |  | 0.163 (0.044) |  | 0.42 (0.371) |
|  | 8h | 0.066 (0.03) |  | 0.011 (0.004) |  | 0.14 (0.062) |  | 0.153 (0.134) |
|  | 24h | 6.647 (3.52) |  | 0.063 (0.057) |  | 0.029 (0.009) |  | 1.787 (1.4) |
|  | 48h | 0.542 (0.341) |  | 0.033 (0.023) |  | 0.007 (0.003) |  | 0.124 (0.072) |
|  |  |  |  |  |  |  |  |  |
| ABA | 4h | 0.01 (0.004) |  | 0.006 (0.003) |  | 0.061 (0.011) |  | 3.136 (1.557) |
|  | 8h | 0.059 (0.035) |  | 0.011 (0.006) |  | 0.116 (0.033) |  | 0.472 (0.355) |
|  | 24h | 0.001 (0.001) |  | 0.035 (0.022) |  | 0.051 (0.034) |  | 1.275 (0.928) |
|  | 48h | 0.002 (0.001) |  | 0.01 (0.005) |  | 0.059 (0.029) |  | 0.423 (0.368) |
|  |  |  |  |  |  |  |  |  |
| SA | 4h | 0.003 (0.001) |  | 0.21 (0.173) |  | 0.34 (0.038) |  | 0.904 (0.639) |
|  | 8h | 0.002 (0.001) |  | 1.901 (0.022) |  | 0.271 (0.104) |  | 0.009 (0.005) |
|  | 24h | 0.001 (0.000) |  | 4.641 (1.367) |  | 0.058 (0.018) |  | 0.006 (0.005) |
|  | 48h | 0.001 (0.000) |  | 1.905 (1.705) |  | 0.057 (0.018) |  | 0.009 (0.005) |

| Control | 4h | 0.007 (0.004) |  | 0.012 (0.007) |  | 0.098 (0.029) |  | 0.101 (0.06) |
| --- | --- | --- | --- | --- | --- | --- | --- | --- |
|  | 8h | 0.029 (0.014) |  | 0.003 (0.001) |  | 0.084 (0.027) |  | 0.517 (0.198) |
|  | 24h | 0.003 (0.001) |  | 0.006 (0.003) |  | 0.117 (0.063) |  | 1.135 (0.643) |
|  | 48h | 0.003 (0.002) |  | 0.003 (0.002) |  | 0.032 (0.010) |  | 7.66 (4.874) |
|  |  |  |  |  |  |  |  |  |
| Ethephon | 4h | 0.001 (0.000) |  | 0.238 (0.223) |  | 8.758 (1.441) |  | 0.004 (0.003) |
|  | 8h | 0.000 (0.000) |  | 0.016 (0.004) |  | 3.102 (1.237) |  | 0.001 (0.001) |
|  | 24h | 0.002 (0.002) |  | 7.814 (7.663) |  | 4.872 (0.144) |  | 0.009 (0.004) |
|  | 48h | 0.000 (0.000) |  | 0.505 (0.303) |  | 2.138 (0.849) |  | 0.222 (0.208) |

**Table S3.** Levels of phytohormones (SE) in *Brassica rapa* shoots and roots in response to hormonal application. The levels of jasmonic acid (JA), abscisic acid (ABA) and salicylic acid (SA) were measured in the treated organ at 4, 8, 24 and 48h after methyl jasmonate (MeJA), ABA, SA or ethephon application (n = 3-4 per treatment and harvest time, except for ABA-treated roots at 24h where n = 2). Due to differences in the methodology of ethephon application compared to the other treatments, a different set of plants was used as control for the ethephon treatment

| **Treatment** | **Time** | **Shoot phytohormone levels**  **(ng mg fresh mass ^-1^)** | | |  | **Root phytohormone levels**  **(ng mg fresh mass ^-1^)** | | |
| --- | --- | --- | --- | --- | --- | --- | --- | --- |
|  |  | **JA** | **ABA** | **SA** |  | **JA** | **ABA** | **SA** |
| Control | 4h | 0.002 (0.000) | 0.02 (0.002) | 0.01 (0.002) |  | 0.062 (0.012) | 0.005 (0.002) | 0.108 (0.041) |
|  | 8h | 0.004 (0.002) | 0.02 (0.002) | 0.055 (0.006) |  | 0.098 (0.015) | 0.00001 (0.000) | 0.057 (0.009) |
|  | 24h | 0.001 (0.000) | 0.011 (0.001) | 0.008 (0.003) |  | 0.089 (0.012) | 0.011 (0.006) | 0.025 (0.003) |
|  | 48h | 0.001 (0.000) | 0.03 (0.002) | 0.006 (0.001) |  | 0.130 (0.019) | 0.028 (0.001) | 0.024 (0.009) |
|  |  |  |  |  |  |  |  |  |
| MeJA | 4h | 0.383 (0.082) | 0.009 (0.001) | 0.007 (0.002) |  | 3.923 (0.623) | 0.006 (0.001) | 0.052 (0.011) |
|  | 8h | 0.198 (0.061) | 0.011 (0.002) | 0.018 (0.006) |  | 3.978 (0.741) | 0.002 (0.001) | 0.019 (0.003) |
|  | 24h | 0.013 (0.002) | 0.009 (0.002) | 0.011 (0.002) |  | 11.728 (0.963) | 0.012 (0.003) | 0.032 (0.009) |
|  | 48h | 0.007 (0.001) | 0.018 (0.004) | 0.005 (0.001) |  | 2.522 (0.456) | 0.003 (0.000) | 0.066 (0.026) |
|  |  |  |  |  |  |  |  |  |
| ABA | 4h | 0.003 (0.000) | 0.259 (0.02) | 0.009 (0.003) |  | 0.106 (0.011) | 0.156 (0.028) | 0.027 (0.005) |
|  | 8h | 0.004 (0.001) | 0.134 (0.01) | 0.015 (0.004) |  | 0.105 (0.019) | 0.214 (0.058) | 0.066 (0.015) |
|  | 24h | 0.002 (0.001) | 0.044 (0.002) | 0.01 (0.005) |  | 0.147 (0.030) | 0.077 (0.003) | 0.078 (0.001) |
|  | 48h | 0.003 (0.001) | 0.036 (0.003) | 0.006 (0.001) |  | 0.105 (0.015) | 0.056 (0.016) | 0.037 (0.023) |
|  |  |  |  |  |  |  |  |  |
| SA | 4h | 0.002 (0.000) | 0.01 (0.002) | 16.727 (1.446) |  | 0.072 (0.031) | 0.006 (0.003) | 7.542 (1.437) |
|  | 8h | 0.002 (0.000) | 0.015 (0.003) | 8.94 (2.182) |  | 0.071 (0.017) | 0.004 (0.003) | 7.950 (0.119) |
|  | 24h | 0.001 (0.000) | 0.007 (0.002) | 0.478 (0.178) |  | 0.107 (0.014) | 0.001 (0.001) | 14.224 (2.657) |
|  | 48h | 0.001 (0.000) | 0.014 (0.004) | 1.373 (0.417) |  | 0.103 (0.013) | 0.001 (0.001) | 2.298 (0.597) |

**Table S3.** (Continued)

| **Treatment** | **Time** | **Shoot phytohormone levels**  **(ng mg fresh mass ^-1^)** | | |  | **Root phytohormone levels**  **(ng mg fresh mass ^-1^)** | | |
| --- | --- | --- | --- | --- | --- | --- | --- | --- |
|  |  | **JA** | **ABA** | **SA** |  | **JA** | **ABA** | **SA** |
| Control | 4h | 0.007 (0.002) | 0.007 (0.001) | 0.022 (0.015) |  | 0.114 (0.016) | 0.004 (0.001) | 0.142 (0.068) |
|  | 8h | 0.007 (0.002) | 0.008 (0.001) | 0.016 (0.011) |  | 0.078 (0.037) | 0.011 (0.004) | 0.095 (0.039) |
|  | 24h | 0.004 (0.001) | 0.005 (0.001) | 0.014 (0.006) |  | 0.085 (0.016) | 0.008 (0.004) | 0.034 (0.007) |
|  | 48h | 0.003 (0.001) | 0.006 (0.001) | 0.008 (0.000) |  | 0.087 (0.008) | 0.026 (0.012) | 0.045 (0.003) |
|  |  |  |  |  |  |  |  |  |
| Ethephon | 4h | 0.005 (0.003) | 0.009 (0.002) | 0.016 (0.008) |  | 0.116 (0.028) | 0.002 (0.000) | 0.103 (0.044) |
|  | 8h | 0.004 (0.001) | 0.011 (0.001) | 0.009 (0.001) |  | 0.080 (0.007) | 0.003 (0.001) | 0.035 (0.006) |
|  | 24h | 0.003 (0.001) | 0.013 (0.003) | 0.018 (0.007) |  | 0.123 (0.012) | 0.002 (0.000) | 0.034 (0.007) |
|  | 48h | 0.002 (0.000) | 0.007 (0.001) | 0.004 (0.000) |  | 0.098 (0.013) | 0.008 (0.004) | 0.202 (0.094) |

**Table S4.** F- and P-values of a two-way ANOVA model on gene expression levels in *Brassica* *rapa* shoots and roots. The ANOVA model contained treatment (control, MeJA, ABA and SA), time (4, 8, 24, 48 h) and their interaction term as factors (n = 3-4 per treatment and harvest time). The expression levels of *VSP2* (*VEGETATIVE STORAGE PROTEIN 2*), *PR1* (*PATHOGENESIS*-*RELATED* *PROTEIN* 1), *ERF1* (*ETHYLENE RESPONSE FACTOR 1*) and *BrLEA4* (*Brassica* *rapa* 18 kDa seed maturation protein-like) were measured in the treated organ after methyl jasmonate (MeJA), abscisic acid (ABA) or salicylic acid (SA) application

| **Gene** | **Shoots** | | |  | **Roots** | | |
| --- | --- | --- | --- | --- | --- | --- | --- |
|  | **Treatment** | **Time** | **Interaction treatment x time** |  | **Treatment** | **Time** | **Interaction treatment x time** |
|  |  |  |  |  |  |  |  |
| *VSP2* | F_3,48_ = 14.66  P < 0.001 | F_3,48_ = 3.78  P = 0.016 | F_9,48_ = 0.43  P = 0.909 |  | F_3,39_ = 35.18  P < 0.001 | F_3,39_ = 3.67  P = 0.02 | F_9,39_ = 3.63  P = 0.002 |
|  |  |  |  |  |  |  |  |
| *PR1* | F_3,43_ = 3.99  P = 0.014 | F_3,43_ = 1.48  P = 0.234 | F_9,43_ = 2.00  P = 0.063 |  | F_3,42_ = 21.83  P < 0.001 | F_3,42_ = 1.67  P = 0.189 | F_9,42_ = 1.37  P = 0.231 |
|  |  |  |  |  |  |  |  |
| *ERF1* | F_3,46_ = 1.81  P = 0.159 | F_3,46_ = 1.89  P = 0.145 | F_9,46_ = 0.66  P = 0.739 |  | F_3,37_ = 6.35  P = 0.001 | F_3,37_ = 16.02  P < 0.001 | F_9,37_ = 2.05  P = 0.061 |
|  |  |  |  |  |  |  |  |
| *BrLEA4* | F_3,43_ = 7.97  P < 0.001 | F_3,43_ = 10.48  P < 0.001 | F_9,43_ = 2.01  P = 0.061 |  | F_3,46_ = 11.22  P < 0.001 | F_3,46_ = 5.93  P < 0.001 | F_9,46_ = 4.16  P < 0.001 |

**Table S5.** F- and P-values of a two-way ANOVA model on the phytohormone levels in *Brassica* *rapa* shoots and roots. The ANOVA model contained treatment (control, MeJA, ABA and SA), time (4, 8, 24, 48 h) and their interaction term as factors (n = 3-4 per treatment and harvest time, except for ABA-treated roots at 24h where n = 2). The levels of jasmonic acid (JA), abscisic acid (ABA) and salicylic acid (SA) were measured in the treated plant organ at 4, 8, 24 and 48h after methyl jasmonate (MeJA), ABA or SA application

| **Measured phytohormone** | **Shoots** | | |  | **Roots** | | |
| --- | --- | --- | --- | --- | --- | --- | --- |
|  | **Treatment** | **Time** | **Interaction treatment x time** |  | **Treatment** | **Time** | **Interaction treatment x time** |
|  |  |  |  |  |  |  |  |
| JA | F_3,43_ = 91.98  P < 0.001 | F_3,43_ = 19.34  P < 0.001 | F_9,43_ = 6.54  P < 0.001 |  | F_3,44_ = 452.2  P < 0.001 | F_3,44_ = 7.68  P < 0.001 | F_9,44_ = 4.11  P = 0.001 |
|  |  |  |  |  |  |  |  |
| ABA | F_3,44_ = 96.07  P < 0.001 | F_3,44_ = 8.8  P < 0.001 | F_9,44_ = 6.92  P < 0.001 |  | F_3,40_ = 26.28  P < 0.001 | F_3,40_ = 2.08  P = 0.118 | F_9,40_ = 3.99  P = 0.001 |
|  |  |  |  |  |  |  |  |
| SA | F_3,43_ = 354.15  P < 0.001 | F_3,46_ = 21.75  P < 0.001 | F_9,43_ = 7.11  P < 0.001 |  | F_3,44_ = 285.06  P < 0.001 | F_3,44_ = 4.31  P = 0.009 | F_9,44_ = 4.49  P < 0.001 |
